# Analysis and Biophysics of Surface EMG for Physiotherapists and Kinesiologists: Toward a Common Language With Rehabilitation Engineers

**DOI:** 10.3389/fneur.2020.576729

**Published:** 2020-10-15

**Authors:** Lara McManus, Giuseppe De Vito, Madeleine M. Lowery

**Affiliations:** ^1^Neuromuscular Systems Laboratory, School of Electrical and Electronic Engineering, University College Dublin, Dublin, Ireland; ^2^Neuromuscular Physiology Laboratory, Department of Biomedical Sciences, University of Padova, Padova, Italy

**Keywords:** rehabilitation, surface electromography, physiotherapy, kinesiology, clinical application, surface EMG tutorial

## Abstract

Recent decades have seen a move toward evidence-based medicine to inform the clinical decision-making process with reproducible findings from high-quality research studies. There is a need for objective, quantitative measurement tools to increase the reliability and reproducibility of studies evaluating the efficacy of healthcare interventions, particularly in the field of physical and rehabilitative medicine. Surface electromyography (sEMG) is a non-invasive measure of muscle activity that is widely used in research but is under-utilized as a clinical tool in rehabilitative medicine. Other types of electrophysiological signals (e.g., electrocardiography, electroencephalography, intramuscular EMG) are commonly recorded by healthcare practitioners, however, sEMG has yet to successfully transition to clinical practice. Surface EMG has clear clinical potential as an indicator of muscle activation, however reliable extraction of information requires knowledge of the appropriate methods for recording and analyzing sEMG and an understanding of the underlying biophysics. These concepts are generally not covered in sufficient depth in the standard curriculum for physiotherapists and kinesiologists to encourage a confident use of sEMG in clinical practice. In addition, the common perception of sEMG as a specialized topic means that the clinical potential of sEMG and the pathways to application in practice are often not apparent. The aim of this paper is to address barriers to the translation of sEMG by emphasizing its benefits as an objective clinical tool and by overcoming its perceived complexity. The many useful clinical applications of sEMG are highlighted and examples provided to illustrate how it can be implemented in practice. The paper outlines how fundamental biophysics and EMG signal processing concepts could be presented to a non-technical audience. An accompanying tutorial with sample data and code is provided which could be used as a tool for teaching or self-guided learning. The importance of observing sEMG in routine use in clinic is identified as an essential part of the effective communication of sEMG recording and signal analysis methods. Highlighting the advantages of sEMG as a clinical tool and reducing its perceived complexity could bridge the gap between theoretical knowledge and practical application and provide the impetus for the widespread use of sEMG in clinic.

## Introduction

Surface EMG is currently an under-utilized clinical tool in rehabilitative medicine, despite its clear potential as a non-invasive measure of muscle activity. It is often considered more complex to analyze than intramuscular EMG, a technique commonly applied in clinical neurology, as parameters of direct clinical relevance cannot be readily extracted (visually or acoustically) from the recorded signal. However, with relatively basic signal processing, important information on muscle activation patterns and muscle properties can be obtained from surface electromyographic (sEMG) signals. This information can potentially provide an objective, quantitative method of assessing muscle function, movement patterns, and local muscle fatigue to inform the clinical decision-making process. Surface EMG features may also provide a more effective means of objectively capturing differences in motor control following surgical or therapeutic interventions, or training and rehabilitation protocols, when compared with more subjective measures based on visual observation, manual palpation, mechanical manipulation, or standard clinical tests. Application of sEMG in terms of real-time feedback can also be used as a tool to help patients gain greater awareness of their own muscle activity and support re-training of movement patterns. Recent developments in wearable sensing technologies enable movement and EMG data to be recorded in more natural environments outside the laboratory setting, during the activities of daily life. These technologies present a range of new opportunities for quantitative assessment and medium to long-term monitoring of movement. However, a basic understanding of signal processing is required to extract or interpret information from EMG or accelerometry signals recorded by the sensors. With smartphones and tablets placing high-powered data sensing and processing capability into the hands of all, and technology becoming an integral part of all professions, it is responsibility of educators and professional bodies to ensure that the next generation of therapists have the technical competency and know-how needed to make the most of this capability within their clinical practice and harness the opportunities it offers.

The speed at which technological innovations are adopted and disseminated is governed by several factors, including the perceived complexity and benefit of the innovation (1, 2). The slow uptake of sEMG as a clinical tool can be largely attributed to the perceived complexity of sEMG and an incomplete understanding of its capabilities and potential among practitioners (and more importantly, among clinical educators). Although these obstacles can be partially overcome through education, the mere availability of tutorials and documented technical information is not enough to encourage the widespread dissemination of sEMG as a clinical tool. A theoretical knowledge of sEMG alone is not sufficient, practitioners need to see how sEMG can benefit everyday practice to be convinced to adopt the technology. The benefit is best demonstrated either by students being taught how to use and apply it by their educators, or though observing it in routine use by experienced practitioners in the clinic. As highlighted by Jette (2), “People follow the lead of other people they know and trust when they decide whether to take up an innovation and change the way they practice.” When correctly presented, the perceived complexity of sEMG can also be broken down, and the technical background needed to accurately record and interpret sEMG signals can be conveyed to students and practitioners in a relatively simple manner. Finally, providing opportunities for practical experience observing and experimenting with sEMG is then critical to bridge the gap from a theoretical knowledge of sEMG to having the ability and motivation to adopt sEMG in clinical practice.

The perception of sEMG as a specialized subject with limited relevance to clinical practice is likely to be first formed during the education and training of practitioners. Physiotherapy and kinesiology education varies considerably across different countries, ranging from an apprenticeship involving clinical or hospital-based training to professional masters or doctoral degree programs (3) (from here on, the term “physiotherapy” will be used to cover “physiotherapy, kinesiology, and physical therapy”). The standard curriculum in tertiary education may cover recording and analysis of sEMG. However, this is not universally the case, and even when taught, these topics are often not covered in sufficient detail to encourage a confident and independent use of sEMG in clinical practice. A gap exists between the theory covered in standard courses and the minimum signal processing and biophysics knowledge and experience required for application of sEMG in clinical practice. These concepts are required to master the recording and analysis methods for sEMG and to ensure the required clinical information can be obtained accurately and reliably. Although there are a number of resources that provide guidelines for sEMG signal processing, this information is sometimes presented using language and terminology that caters to readers with an engineering or technical background. Notable exceptions include the books by Criswell (4), Kamen and Gabriel (5), Barbero et al. (6), and Chapter 8 of Robertson et al. (7) which provide a detailed treatment of sEMG recording and analysis methods targeted specifically at practitioners (a full list of relevant resources targeted at practitioners is available in the “Further Reading” Supplementary Material).

The aim of this paper is to address technical barriers to the widespread adoption of sEMG in the clinic, specifically those related to the perceived complexity and benefit of sEMG. Examples are described to illustrate the wide range of clinical sEMG applications, from simple biofeedback (requiring minimal knowledge of sEMG concepts) to more advanced sEMG signal analysis that can provide additional detail on neuromuscular function (e.g., sEMG median frequency can provide information on muscle fatigue). We then identify key information that is needed to successfully record, process and interpret sEMG signals in a clinical setting, and aim to present it in an accessible way to a non-technical audience. The paper begins with an overview of the physics and physiology underlying the generation of sEMG signals and examples of clinical applications (section 2: Background and Applications). Basic signal concepts including time and frequency domain analyses are introduced in section 3: Basic Signal Concepts. The main factors to consider when choosing equipment and recording EMG signals are then outlined (section 4: EMG Signal Acquisition and Recording) and key topics in signal processing relevant to sEMG analysis explained, i.e., sampling, filtering, and frequency domain analysis (section 5: EMG Signal Pre-Processing and Analysis). The topics covered could be incorporated into the curricula for physiotherapists to provide the foundational knowledge needed to reliably record and interpret sEMG signals to extract clinically meaningful information. Although the material covers topics that may be unfamiliar to readers coming from a non-engineering background, the only pre-requisite to understanding the material is familiarity with basic mathematical concepts, e.g., concept of an equation, sine, logarithm. This allows the material to be more easily understood by readers from a non-technical background when compared to other introductory signal processing texts, which often require a relatively strong mathematical knowledge.

This paper is designed as a tutorial to enable readers to bridge the gap between theory and how it is applied in practice though EMG signal analysis. To promote the practical application of the key concepts covered in this paper, EMG data is available in the Supplementary Material and accompanying MATLAB (requires license) and Octave (free) software code is provided to illustrate different signal processing concepts (https://doi.org/10.5281/zenodo.4001609). These data and software codes could form the basis of a practical tutorial or workshop on sEMG. The code provided can be used as a template to be adapted and used by readers in the analysis of their own signals. Although many sEMG recording systems supply software to provide estimates of signal features, such as sEMG amplitude or frequency content, it is often not clear to the user how these features are calculated. To the non-expert user, the appropriate choice of parameters to extract the signal features of interest may not be apparent. However, enabling users to develop and alter their own code allows them to explore how changing the analysis parameters can affect the features extracted from the sEMG. This empowers the user, providing transparency and the awareness of the processing methods rather than a “black box” type approach, and enables them to tailor their analysis to specific applications. Even when using standard software packages, it is important that users understand how the parameters chosen for signal analysis influence the results obtained.

Finally, by introducing these topics in a way that is accessible and clinically useful, we demonstrate a pathway for incorporating technical and scientific aspects of sEMG recording and analysis into the educational curriculum for physiotherapists. In this way, we aim to reduce technical barriers to the incorporation of surface EMG in clinical applications and provide a bridge between theoretical concepts and practical applications by both reducing the perceived complexity of sEMG and highlighting its benefits as a clinical tool.

## Background and Applications

### EMG Generation

**Figure 1:**
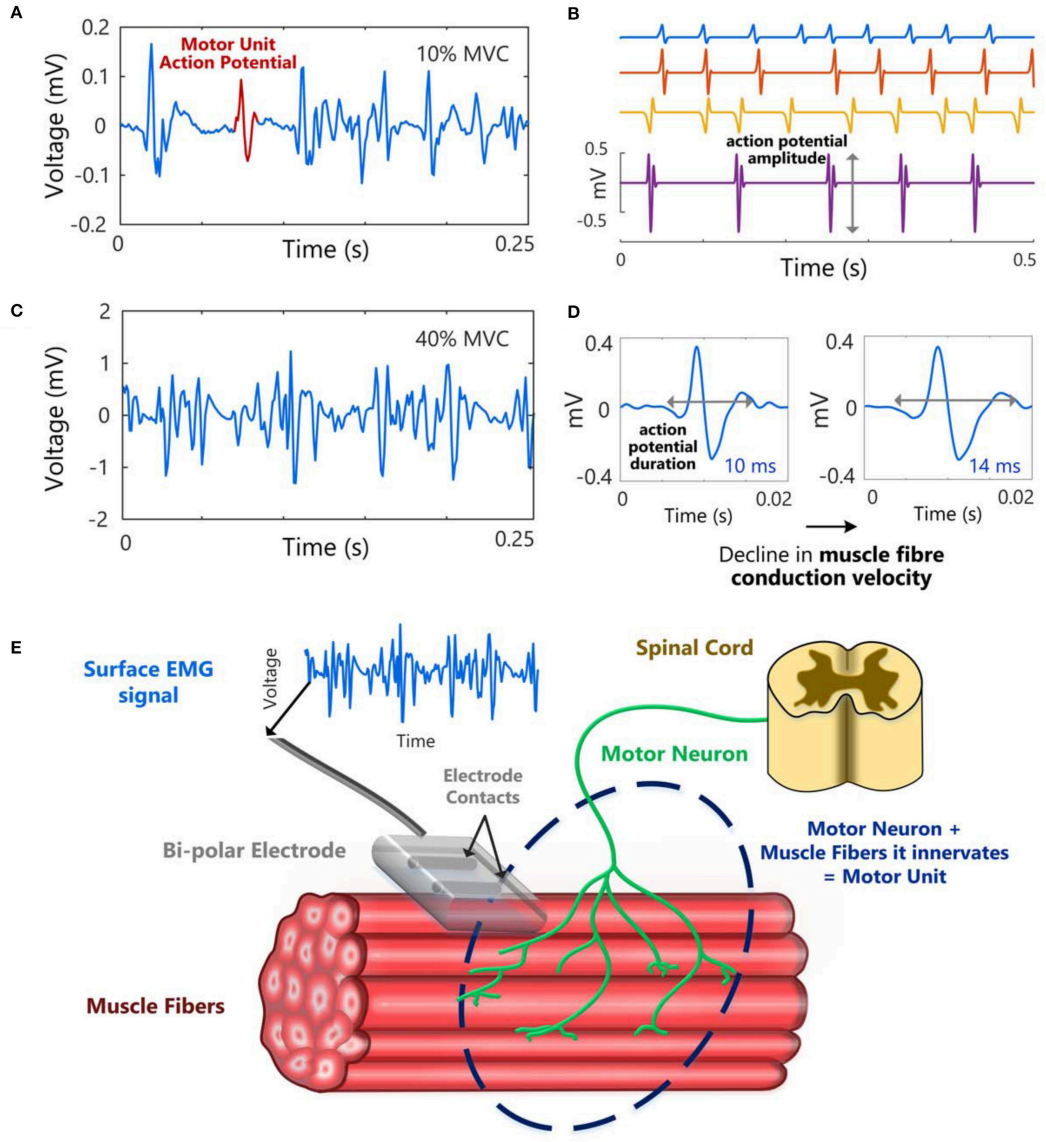
Example of a surface EMG signal at a low force level (10% of maximum voluntary contraction, MVC) **(A)** and a higher force level, 40% MVC **(C)**, in the first dorsal interosseous muscle. **(B)** Single motor unit action potential trains with different inter-spike intervals (ISIs), i.e., different motor unit firing rates and **(D)** an illustration of the increase in action potential duration that can occur with a decline in muscle fiber conduction velocity. **(E)** A schematic to illustrate a motor unit, and how a surface EMG signal could be recorded from a muscle using a bipolar electrode (two electrode contacts).

The EMG signal is the electrical activity generated by a contracting muscle which can be detected by placing an electrode^1^ or pair of electrodes on the skin above the muscle of interest. During muscle activation, there is a flow of charged particles (ions) across the muscle fiber membrane. The rate of flow of charge is called the electrical current (I) and is measured in Amperes (electric charge per second). Electrical currents within the muscle alter the electrical potential in the surrounding tissue. The difference in electrical potential or voltage between two points is measured in Volts (V). The voltage detected at the skin surface is influenced by the resistance or impedance [quantified in Ohms (Ω)] to the flow of electric current provided by the surrounding muscle, subcutaneous tissue, and skin. The time-varying voltage distribution present on the skin surface due to the electrical activity of a muscle is termed the sEMG signal, see Figure 1, and can be used to provide information about the muscle contraction^2^ (see section Practical Applications of Surface EMG in the Clinic). These basic principles of electricity are fundamental to the understanding of the more advanced EMG topics covered in this paper [see also Barry (8) and Kamen and Gabriel (5)].

Signals from the brainstem/spinal cord are transmitted to the muscle by motoneurons. When a motoneuron is activated (i.e., discharges), synaptic transmission at the neuromuscular junction results in a transient change in electrical potential, known as an action potential, across the muscle fiber membrane of each muscle fiber innervated by the motoneuron. The motor unit^3^ action potential refers to the electrical potential recorded due to the activation of the muscle fibers innervated by a motoneuron. The sEMG signal is a summation of action potentials generated by motor units lying within the detection volume of the electrodes, Figure 1A [for detailed accounts of EMG signal generation see De Luca (9), Kamen and Caldwell (10), Moritani et al. (11), and Farina et al. (12)]. Each motor unit action potential (MUAP) waveform will have a distinct shape, Figure 1B, which represents the recorded electrical potential over time. The shape of the action potential will depend on the motor unit properties (e.g., number of muscle fibers innervated by the motoneuron and their cross-sectional area and fiber type) and the location and orientation of its muscle fibers relative to the position of the recording electrodes [for further details on MUAP properties see Barkhaus and Nandedkar (13) and Rodriguez-Falces (14)]. In order to increase the force generated by a muscle, there must be an increase in the firing rates of motor units which are already active and/or additional motor units must be recruited. At very low levels of muscle activation (e.g., <10% of maximum voluntary contraction, MVC) or with intramuscular EMG (detected with needles or wires inserted into the muscle), it is sometimes possible to distinguish individual motor unit action potentials, Figure 1A (ranging in duration from 5 to 30 ms, Figure 1D). At higher muscle contraction levels (e.g., 10–100% MVC), it is rarely possible to visually distinguish individual motor units, as the number of MUAPs contributing to the signal increases. The action potentials from all active motor units sum together generating a random-looking EMG signal at the skin surface, Figure 1C. The firing times of individual motor units can be extracted from sEMG signals recorded with two-dimensional arrays of electrodes using specialized sEMG decomposition algorithms (15–17). This method provides information on the discharge times of individual motoneurons, with a greater yield of detected motor units than can typically be obtained using invasive intramuscular EMG. sEMG decomposition is a specialized topic which will not be covered in this paper, for further details readers are referred to de Luca et al. (18), Drost et al. (19), Stegeman et al. (20), and Farina and Holobar (21).

### Practical Applications of Surface EMG in the Clinic

In the assessment and diagnosis of neuromuscular disorders, EMG is typically invasively recorded within the muscle using needle electrodes (either concentric or monopolar). Intramuscular EMG recordings can isolate single motor unit activity and are used to detect abnormalities in motor unit firing patterns or in action potential shape, and pathological spontaneous activity in relaxed muscles. However, due to the small detection volume of needle electrodes, intramuscular EMG recordings reflect the activity of a small number of motor units whose muscle fibers are located close to the detection site. Moreover, this technique is usually limited to low levels of isometric muscle contraction with a relatively small number of active motor units in order to reliably discriminate or extract the activity of individual motor units. Physiotherapists are often more interested in extracting information on temporal patterns of activity from the muscle as a whole or from groups of muscles, often during functional movement. Surface EMG provides a non-invasive, global measurement of muscle activity, which may be more suitable for applications in movement analysis that require frequent assessments or information on the patterns of activation of multiple muscles (e.g., in sport, rehabilitation, and occupational medicine). Surface EMG can be a valuable tool in both the assessment and treatment of patients and can be used to objectively, and quantitatively, measure progress, and evaluate treatment outcomes.

**Figure 2:**
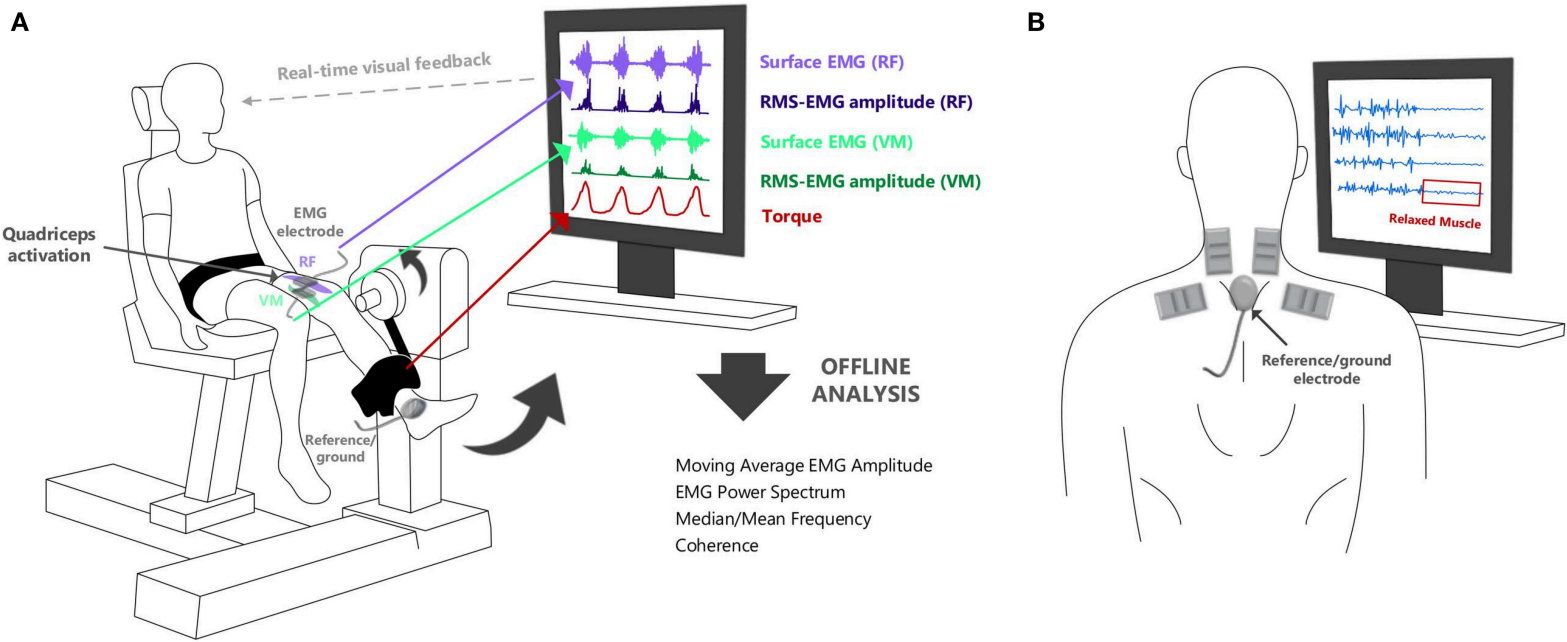
**(A)** Surface EMG can be used to provide real-time feedback of muscle activation during neuromuscular assessments. It can show the relative timing of activation from selected muscles in different tasks. **(B)** Visual biofeedback from surface EMG can also aid in muscle training to ensure that rehabilitation tasks are optimally performed (and the correct muscles are “relaxed” or “activated” as required by the task).

Examples of applications of sEMG signals in the assessment and treatment of patients are summarized in Table 1. Surface EMG recorded from simultaneously active muscles can provide the therapist with information on the symmetry and relative activation of these muscles during different movements. For example, sEMG recorded from the left and right erector spinae muscles during a low back evaluation or from the vastus medialis and lateralis during a patellar subluxation evaluation can be used to determine whether there is balanced activation from paired muscles [Chapter 4 in Schwartz and Andrasik (23)]. Surface EMG can also be used as a therapeutic aid in the treatment of patients and enable them to gain more awareness and control of their own muscle activity. The amplitude of the sEMG signal can be shown to the patient and therapist (i.e., biofeedback) to provide an objective measure of the degree of muscle activation, Figure 2A (24). Surface EMG biofeedback can be used in rehabilitation protocols to help patients self-regulate elevated muscle activity, strengthen/train weak, inhibited or paretic muscles, and facilitate a reduction in tone in a spastic muscle (25). Schwartz and Andrasik (23) illustrate several applications of sEMG biofeedback, including an example in patients with shoulder problems. In this example, it is suggested that sEMG could be recorded from the upper and lower trapezius during shoulder abduction and a virtual channel constructed to display the relative activation of the lower trapezius (i.e., amplitude of the lower trapezius sEMG divided by the sum of the amplitudes of the upper and lower trapezius sEMG). This channel could be displayed to the patient visually to target the lower trapezius (increase recruitment), which can become inhibited and limit full range of motion in shoulder abduction. In a similar way, the inclusion of EMG biofeedback in conventional exercise programs can facilitate recovery after surgery (26, 27) and can also improve the effectiveness of training programs [e.g., pelvic floor muscle training (28–30)]. Surface EMG biofeedback can also be useful in cases where muscular tension causes pain, as the visual stimulus can help the patient to relax or deactivate the muscles [e.g., alleviating neck (31, 32) and low back pain (33)], Figure 2B. Muscle activation can be assessed using sEMG during different exercises in order to identify abnormal patterns of activation, and aid in locating the source of chronic pain (34). Visual feedback on muscle activation has also been shown to improve gait quality in both hemiplegic patients and in children with cerebral palsy (35, 36).

**Table 1:** Examples of sEMG applications in assessment and treatment, see Chapter 10 in Criswell (4).

**Assessment**	
**Assess level of muscle activation:**	**sEMG evaluation:**
• Unwanted muscle activation during rest	• Evidence of MU activity in baseline sEMG recording
• Relative activation of different muscles across the range of motion^a^	• Variation in sEMG amplitude across the range of motion^a, b^
• Presence of unwanted muscle activation/inhibition during muscle contractions	• Excessive/insufficient muscle activation (higher/lower than expected sEMG amplitude) relative to the task or in relation to other synergists^b, c^
• Inappropriate muscle co-activation during bilateral movements	• Differences in sEMG activity between homologous muscles on the involved and uninvolved sides during bilateral movements^b, c^
**Assess timing of muscle activation:**	**sEMG evaluation:**
• Altered recruitment/derecruitment of muscles during eccentric and concentric phases of movement • Inappropriate timing of agonist/antagonist muscle activation during joint stabilization	• Delayed muscle onset during movement • Premature muscle onset during movement • Muscle active for an excessive (or insufficient) time period during movement
**Assess muscle fatigue:**	**sEMG evaluation:**
• Indirect estimate of changes in muscle fiber conduction velocity (MFCV) associated with peripheral fatigue	• Rate of decline of the mean/median frequency of the sEMG signal (see section Surface EMG Spectral Features (Frequency Domain)) during a fatiguing contraction and subsequent recovery (normalized to baseline value)
• “Global” estimate of MFCV	• Estimated from the delay between sEMG signals from spatially displaced electrodes
• Changes in muscle activation (motor unit firing rate/recruitment) to compensate for reduced force generating capacity	• Increase in sEMG amplitude with respect to baseline value
**Treatment**	
**Objective:**	**Use of sEMG:**
• Uptraining muscle(s) (i.e., increase sEMG amplitude)	• sEMG activity can be provided as feedback to the patient as an aid to increase awareness of their level of muscle activation • Begin with training an isolated muscle, recording also from other muscles to ensure they are not inappropriately recruited to the contraction • Threshold level can be set a for sEMG activation and patient encouraged to exceed this threshold • Threshold can be gradually increased to encourage patients to increase the strength of the muscle contraction
	• A threshold could also be used in endurance training, where the subject must maintain a target level of muscle activation
• Relaxation or down-training muscles (i.e., reduce sEMG amplitude)	• Record from muscles that are chronically hyperactive to promote relaxation • Threshold can set be for muscle activation and encourage patient to relax the muscle to keep below this threshold • This threshold can be gradually lowered over time to increase relaxation ability

a *Resources are available showing the normal template for muscle activations during different movements, for example the atlas in Part III of CRAM's provides a “benchmark” for the relative activation of certain muscles during static load conditions (e.g., during normal shoulder abduction, there should be a balanced activation of the upper and lower trapezius)*.

b *Caution should be exercised when interpreting sEMG amplitude due to intersubject and intrasubject variability associated with factors such as electrode contact, electrode placement, anatomical factors, temperature etc. which can influence the signal amplitude*.

c *Normalization of sEMG amplitude with respect to a reference value is usually recommended, see Besomi et al. (22). However, sEMG signals between homologous muscle groups can be approximately compared (e.g., between left and right upper trapezius)*.

Surface EMG can also offer insights into diseases of the central nervous system which can affect the regulation and coordination of movement across the body. This can manifest as a reduction in (paresis/weakness), or loss of (paralysis), the desired motor output. Other diseases of the central nervous system can cause involuntary movements of the body (e.g., chorea, dystonia, seizures in epilepsy, tremor in Parkinson's disease, and essential tremor). Surface EMG offers a non-invasive alternative to intramuscular EMG in the detection of involuntary muscle twitches arising from spontaneous motor unit activity (i.e., fasciculation potentials) (37, 38). Surface EMG recordings are also commonly used in rehabilitation and biomechanics to investigate how movement is coordinated between multiple muscles during different tasks (e.g., during rest, gait, and fine hand movements) with the aim of differentiating between normal and pathological motor control in different conditions or identifying changes in response to interventions such as exercise or when using a device such as an exoskeleton. The amplitude of the sEMG signal can be used to examine the timing of muscle activity and the relative intensity or interaction between simultaneously active muscles, see section Surface EMG Amplitude Features (Time Domain), Table 1, Figure 2A.

In addition to providing information on muscle activation, in certain conditions sEMG can also be used to indirectly monitor changes in muscle force and in the underlying muscle fiber conduction velocity (MFCV). During isometric^4^ muscle contractions, the sEMG amplitude can be used to infer information about muscle force [with important caveats, see de Luca (39)]. Surface EMG recordings can be combined with accelerometry or force data to provide a more complete picture of muscle function during different motor tasks. Simultaneous sEMG and muscle force recordings during isometric muscle contractions may provide a more objective method of assessing local muscle fatigue in the clinic when compared with subjective mechanical techniques. Changes in the amplitude and the mean/median frequency of the sEMG signal can also provide insight into the relative prevalence of central^5^ and peripheral fatigue^6^ in neuromuscular disorders (40). An inability to sustain a voluntary, submaximal muscle contraction combined with a minimal decrease in the sEMG median frequency could indicate that the impairment is central in origin, arising from a suboptimal voluntary drive from brain to muscle. The mean or median frequency of the sEMG signal provides an indirect assessment of changes in muscle fiber conduction velocity, however, a more direct, “global” estimate of muscle fiber conduction velocity (MFCV) can also be calculated using sEMG [estimated as the time taken for a sEMG signal to travel between two spatially displaced recording electrodes, (41, 42)]. This technique to estimate MFCV can be applied proficiently even during the execution of highly dynamic movements [e.g., cycling Farina et al. (43) and Sbriccoli et al. (44)]. Surface EMG has been used to assess differences in MFCV in several neuromuscular disorders (e.g., Duchenne muscular dystrophy, myotonic dystrophy) (38).

More recently, non-linear methods, such as recurrence quantification analysis and entropy, have been used to characterize the degree of similarity and repeating structure within sEMG signals (45, 46), see reviews by Clancy et al. (47) and Mesin et al. (48). These non-linear features have been shown to capture differences in sEMG signals under conditions where normal motor unit synchronization is enhanced, e.g., during muscle fatigue and in subjects with Parkinson's disease (49–52). Lastly, sEMG can be used in conjunction with electrical stimulation to record the amplitude of a compound muscle action potential^7^ (CMAP). The CMAP amplitude is used to diagnose neuromuscular transmission disorders and can also be used to estimate the number of functional motor units within a muscle [for a detailed account of clinical applications see Zwarts et al. (53)].

In recent years, sEMG electrode arrays or grids have become more widely used, offering several advantages over traditional/conventional sEMG recordings. Electrode arrays can be used to locate the innervation zone of a muscle, accurately estimate muscle fiber conduction velocity, map motor unit action potential propagation across a muscle, and inform on the length and orientation of the muscle fibers. Using high density sEMG arrays the sEMG signal can be sampled across different points above the muscle, so that the spatial distribution of the sEMG signal can also be analyzed. Surface EMG recordings from electrode arrays can be decomposed using specialized algorithms to provide information on single/individual motor unit activities. Though sEMG arrays currently have limited clinical use, they offer significant potential for applications in neurology to monitor changes in the characteristics of the motor unit action potential waveform and motoneuron firing patterns in different neuromuscular disorders. However, a main drawback of sEMG array recordings is that they are more complex to analyze and interpret, requiring specialized algorithms to extract individual motor unit activities, and to assess the accuracy of the detected motor unit firing trains. For many applications in sport and rehabilitative medicine, much of the required information can be obtained from traditional bipolar sEMG (e.g., assessment of muscle activation and fatigue). The placement of conventional sEMG electrodes is relatively straightforward and recorded sEMG signals can be successfully processed and analyzed with some basic knowledge of signal processing (with the key topics outlined in this paper). The varied clinical applications of sEMG are discussed in detail in Kamen and Gabriel (5), Criswell (4), and Barbero et al. (6), Merletti and Farina (54) and in Chapter 8 of Robertson et al. (7).

## Basic Signal Concepts

### Description of a Signal

**Figure 3:**
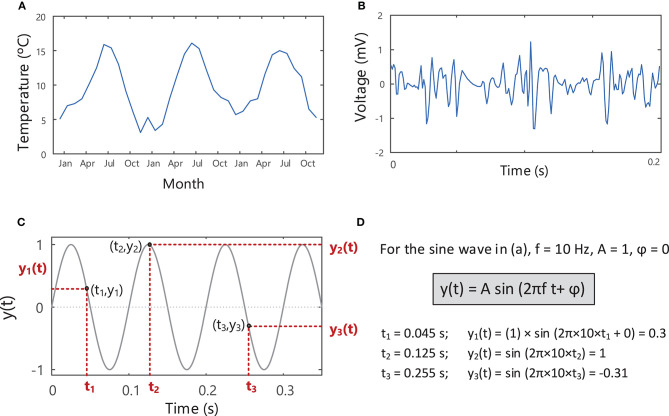
Signals describing the variation of a measurable quantity over time, e.g., **(A)** temperature and **(B)** voltage. **(C)** A sine wave with an amplitude of 1, frequency of 10 Hz and phase of zero, showing the variation in the signal over time. The instantaneous value of the sine wave, y(t), shown in **(C)** can be found at each point in time, t, using the equation in **(D)**. See Example (i) in Tutorial Code.

Any physical quantity (e.g., temperature, voltage, current) that varies over time can be described as a signal and can be represented visually on a graph depicting its waveform or pattern over time, Figures 3A,B. A simple sine wave is described by three characteristics: amplitude (*A*), frequency (*f*), and phase (ϕ), Figure 3D.

**Figure 4:**
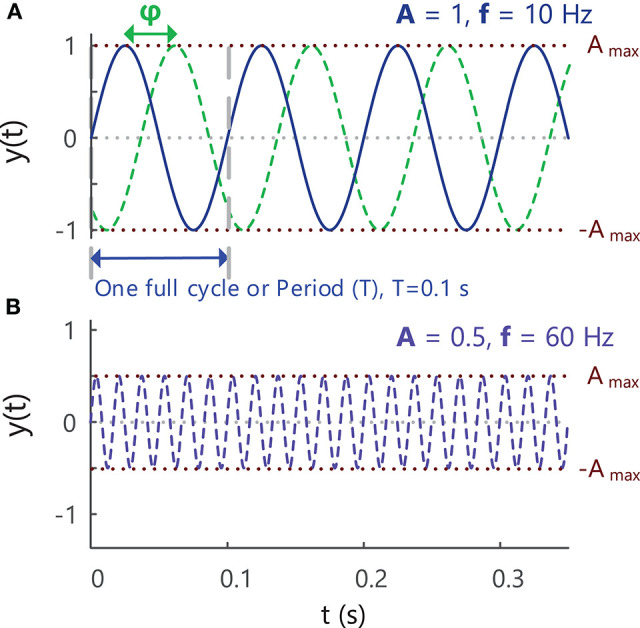
**(A)** A sine wave with an amplitude of 1, frequency of 10 Hz and phase of zero, showing the variation in the signal over time. A second sine wave with the same amplitude and frequency but different phase (–π/2 = −90°) is indicated with the green dashed lines. As the value of a sinusoidal signal at any point in time is based on circular motion, the phase of a signal is expressed as an angle in radians or degrees (start of period = 0°, end of period = 360° or 2π radians). **(B)** A sine wave with an amplitude of 0.5, frequency of 60 Hz and phase of zero. See Example (ii) in Tutorial Code.

Sine waves are periodic signals, which means that they repeat in time after a period of *T* seconds (s), with a frequency of *1/T* “cycles per second” or Hertz (Hz), Figure 4. The period and frequency of a waveform are inversely related to one another ($f=\frac{1}{T}$), so as the period of a waveform decreases, its frequency will increase and vice versa. Sine waves can have different frequencies and amplitudes and can also be shifted in time relative to one another, i.e., have different phases, Figure 4. Sine waves of different frequencies and amplitudes are created in Example ii in Tutorial Code in the Supplementary Material (https://doi.org/10.5281/zenodo.4001609).

### Frequency Domain Analysis

**Figure 5:**
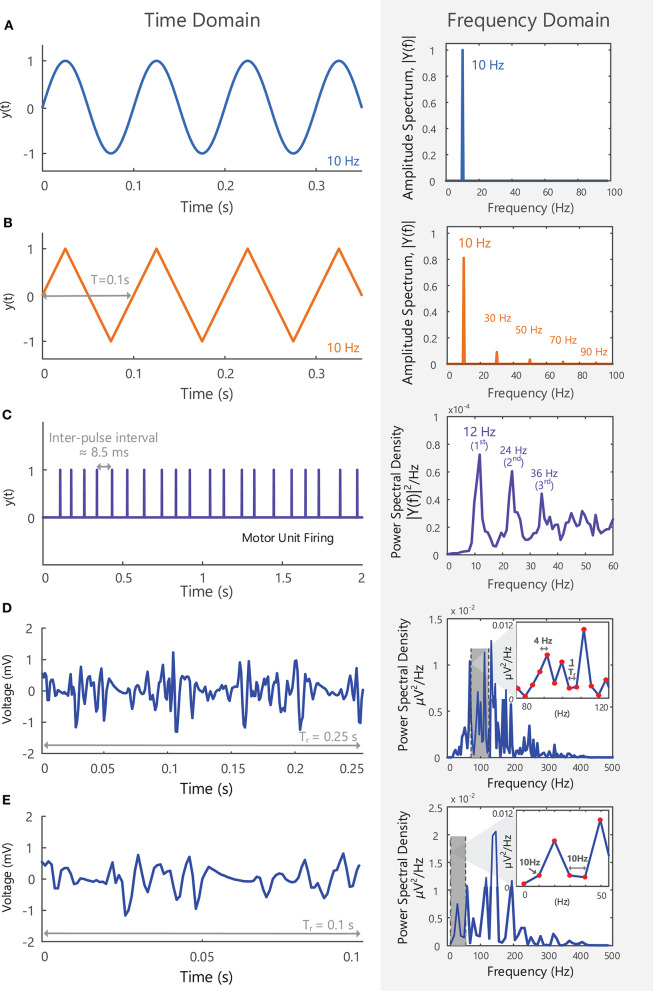
**(A)** A 10 Hz sine wave with an amplitude of 1, shown in the time domain, y(t), and in the frequency domain, Y(f), after applying a Fourier Transform. All the power within the signal is contained at a single frequency (i.e., fundamental frequency or first harmonic−10 Hz). **(B)** A triangle wave with a repetition rate of 10 Hz shown in the time and frequency domain, y(t) and Y(f), respectively. As a non-sinusoidal wave, it contains frequency components at multiples of the first harmonic (note: triangle waves contain only odd harmonics). See Example (iii) in Tutorial Code. **(C)** The firing of a single motor unit over 2 s, shown in the time and frequency domain. The motor unit fires at an average frequency of 12 Hz (fundamental frequency of the spike train), but spectral peaks at multiples of 12 Hz can be observed in the frequency domain. **(D)** A 0.25 s EMG signal in the time and frequency domain. The length of the signal determines the frequency resolution (1/T_r_ = 4 Hz) and the lowest frequency that can be detected in the frequency domain (4 Hz). **(E)** A 0.1 s EMG signal is too short to observe frequencies lower than 10 Hz and can only detect frequency components that are multiples of 10 Hz.

Signals can be represented in the time domain, Figures 3, 4, or can be transformed to the frequency domain by applying the Fourier Transform^8^. Time domain analysis of EMG signals can be used to identify when a muscle is “active” or “inactive” or to provide information on the relative level of muscle activation [section Surface EMG Amplitude Features (Time Domain)]. Applying the Fourier transform to EMG signals provides information on how the signal power is distributed across different frequencies, i.e., it allows the signal to be examined in the frequency domain [section Surface EMG Spectral Features (Frequency Domain)]. The Fourier Transform works on the principle that any periodic signal can be represented as a sum of sine and cosine waves of different amplitudes, frequencies, and phases. The amplitude (*A*) or power of each sinusoidal component can be examined as a function of frequency, providing the amplitude and power spectrum of the signal, respectively, Figure 5. The area under the power spectrum (measured in *V*^2^)^9^ corresponds to the total energy contained within the signal.

For a perfect sine wave, all the energy in the signal is contained at one frequency (the fundamental frequency), Figure 5A. More complex signals, such as electroencephalographic (EEG) or EMG signals, have a broad frequency content with signal power distributed across a range of frequencies. Most of the sEMG signal power is contained between 10 and 400 Hz, Figures 5D,E, with frequency components outside of this bandwidth primarily due to noise at the electrode-skin interface and electrical interference. Higher frequency components up to 5,000 Hz can be observed in intramuscular EMG signals.

The length of the signal segment or epoch (*T*_*r*_) determines the “frequency resolution” of the Fourier-transformed signal and the lowest detectable frequency component (*1*/*T*_*r*_). For example, when 0.25 s of the sEMG recording in Figure 5D is examined in the frequency domain it will have a frequency resolution of 1/0.25 Hz = 4 Hz. Similarly, a 0.1 s sEMG recording will have a frequency resolution of 10 Hz (its frequency components will be 10 Hz apart), Figure 5E. It will thus be too short in duration to detect frequency components below 10 Hz, or frequencies lying between 10 and 20 Hz, 20 and 30 Hz, and so on.

## EMG Signal Acquisition and Recording

Careful skin preparation and choice of electrode type, electrode placement and recording configuration, including filter settings and amplifier gain, are essential to record high quality EMG signals with low noise. Key information needed to optimize the quality of recorded sEMG signals is summarized below, and more detailed information on EMG instrumentation standards can be found in Tankisi et al. (55), Gitter and Stolov (56), Gitter and Stolov (57), and Merletti and Cerone (58).

### Skin Preparation

One of the most important steps in optimizing the quality of sEMG recording is the preparation of the skin surface before electrode placement. The skin should be exfoliated to remove dead skin cells, shaved if necessary and cleansed (if an abrasive gel is used for exfoliation, this should be removed before electrode placement). A conductive gel can be rubbed on the skin (and then removed from the surface, to avoid short-circuiting^10^ the electrode contacts) or placed on the electrode contacts. These steps reduce the electrode impedance^11^ and ensure it is similar across all electrode contacts. The interface or contact point between the electrode and skin generates random noise, part of which can be reduced by reducing the electrode impedance. This noise occurs due to the change in current carrier at the electrode-skin interface, from ion^12^ current (human body) to electron current (electrode).

### Choice of Electrode

There are two main configurations of sEMG electrode: traditional bipolar sEMG and multi-channel sEMG arrays or grids. Bipolar surface electrodes are simple to apply, and relevant parameters are relatively easy to extract from the recorded EMG signals to give a general overview of the muscle activation. More detailed information can be extracted from sEMG recorded with electrode arrays or grids (e.g., identify innervation zone of the muscle, measure action potential propagation velocity, assess the distribution of motor unit activity across a region of the muscle, detect single motor unit activity). However, the procedure for recording and analyzing sEMG data from electrode grids is more complex, and specialized decomposition algorithms are required to extract the firing times of individual motor units. Considerations for the choice of electrode (both surface and intramuscular) are outlined in detail in Soderberg and Knutson (59) and Besomi et al. (60).

**Figure 6:**
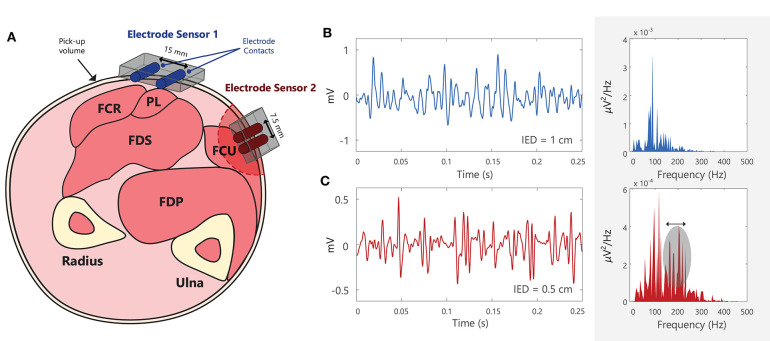
**(A)** Schematic of the cross-section of the forearm, with the approximate locations of different muscles: flexor carpi radialis (FCR), palmaris longus (PL), flexor digitorum superficialis (FDS), flexor digitorum profundus (FDS), and flexor carpi ulnaris (FCU) (62). Note that electrode contacts should be placed approximately parallel to the muscle fiber direction. Electrode sensor 1 is placed over PL, but it will detect muscle activity (or crosstalk) from the adjacent/deep muscles, FCR and FDS. Electrode sensor 2 has a smaller inter-electrode distance (IED) than Electrode sensor 1 and will thus have a smaller pick-up volume. **(B,C)** Two EMG signals recorded using different electrodes with different IEDs, shown in both the time and frequency domains. EMG signals recorded using smaller IEDs can capture more high frequency components when compared with larger IEDs. Note that this diagram is for illustrative purposes and that the power spectra of the EMG signals cannot be directly compared between **(B)** and **(C)**, as they were recorded in different muscles, under different conditions, using different electrodes. See Example (xi) in Tutorial Code.

The selectivity of sEMG electrodes is determined by the distance between electrode contacts (inter-electrode distance, IED), and the area of the detection surface (contact area between electrode and skin surface), see Example (xi) in Tutorial Code. The sEMG recorded by the electrode is the average of the voltage at the skin surface underneath the electrode contact (61). Large electrodes will thus introduce more “averaging” of the EMG signal. The detection volume of the electrode will be greater for larger IEDs, Figure 6A. When recording EMG from small superficial muscles (close to the skin surface), or from muscles with a small surface area beneath the overlying skin, the selectivity of the recording electrode can be increased by decreasing the IED. Orientating the electrode along the direction of the muscle fibers further increases the selectivity of the EMG recording. In applications requiring selective recording of small muscles, larger IEDs with large pick-up volumes may detect unwanted EMG signals (or cross-talk) from muscles other than the muscle of interest (63). In these cases, intramuscular or wire EMG may be preferred to provide a more selective EMG recording (64). Recordings from electrodes above large amounts of subcutaneous fat tissue are more susceptible to cross-talk from surrounding muscles (65, 66). The SENIAM (Surface ElectroMyoGraphy for the Non-Invasive Assessment of Muscles) guidelines make a general recommendation of 10 mm for the electrode contact diameter and IED of 20 mm for bipolar electrodes (i.e., 2 electrode contacts) (67), however, these will vary according to the experimental goals. Barbero et al. (6) and Criswell (4) present comprehensive atlases outlining the correct placement for an electrode pair (bipolar electrode) when recording sEMG from a range of different muscles [the Atlas in Barbero et al. (6) describes the innervation zones of 43 muscles]. For electrode arrays or grids the electrode contact diameter should generally be < 5 mm and the IED < 10 mm in order to effectively sample the EMG signal across the skin surface (68). Importantly, the selected IED will determine the frequency components that can be detected and the bandwidth of the recorded EMG signal, Figures 6B,C. Smaller IEDs used in sEMG grids enable higher frequency signal components to be captured, Figure 6B, making it easier for decomposition algorithms to discriminate the action potential waveforms of different motor units.

Information on recommended electrode placement procedures for different muscles and muscle areas can also be found in the SENIAM guidelines (http://seniam.org/sensor_location.htm). Typically, the preferred location of the electrode is on the midline of the muscle, midway between the nearest innervation zone^13^ and the myotendinous junction (39).

### Choice of Amplifier

EMG signals must be amplified (increasing the signal amplitude) and filtered before they are sampled and stored for processing. In order to reach the amplitude required for signal sampling and conversion from analog to digital format (i.e., going from a continuous to a discrete/sampled signal), surface EMG signals must be amplified (typically by a factor of 1,000–1,500, i.e., raw sEMG signals are in the range of microvolts and are amplified to the range of millivolts). In active electrodes, the pre-amplifier is located on or within the electrode itself, **Figure 10B**, rather than in an external circuit (as in passive electrodes). The use of active electrodes reduces the amount of signal noise being amplified, which is important in experiments where there is a high likelihood of EMG signal contamination from motion artifact (e.g., sEMG recorded during movement or exercise). Motion artifact can be further reduced with wireless electrodes.

**Figure 7:**
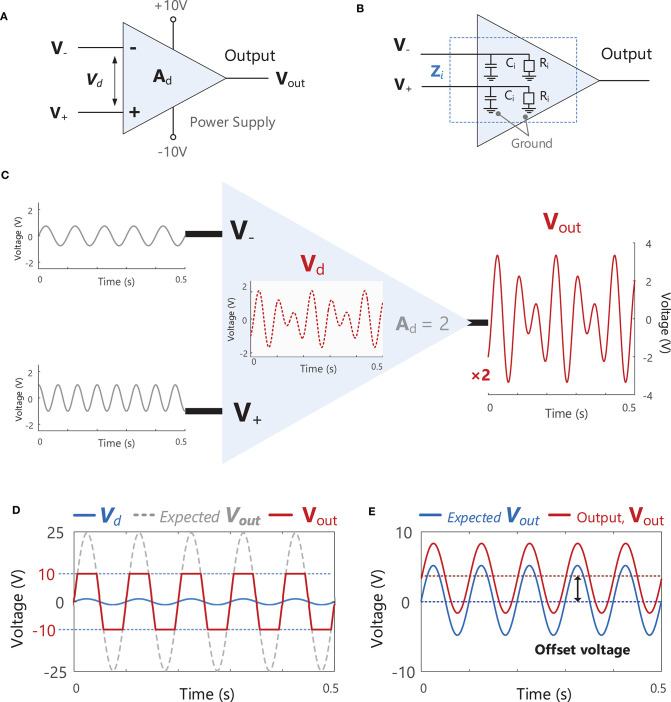
**(A)** A schematic of an ideal differential amplifier with two inputs V_−_ and V_+_, an output V_out_ and power supply connections (+10 and −10 V). **(B)** A schematic of the internal input impedance, Z_i_. present at both amplifier inputs V_−_ and V_+_. Z_i_ consists of a resistive component, R_i_, and a capacitive component, C_i_. **(C)** A schematic illustration the function of a differential amplifier, which receives two input signals at V_−_ and V_+_, calculates the difference between these signals, V_d_ (V_d_ = V_+_ - V_−_), and multiplies (amplifies) V_d_ by the gain of the amplifier, A_d_ (in this example the signal is increased by a factor of 2). See Example (xii) in Tutorial Code. **(D)** If the gain of the amplifier is increased to a level where the expected V_out_ exceeds the level of the power supply voltage (e.g., ± 10 V in the amplifier shown in **A**), the actual V_out_ will be “clipped” or “limited” at the power supply voltage. **(E)** An example of an offset voltage being present in the amplifier output, V_out_.

Bioelectric or biological amplifiers, such as those used in EMG recording, Figures 7A,B, are usually differential amplifiers. This means that they amplify the voltage difference (*V*_*d*_ = *V*_+_ - *V*_−_) between two points (electrodes) by a factor of A_d_ (the amplifier differential gain) to obtain the output voltage, *V*_*out*_, Figure 7C (i.e., *V*_*out*_ = *A*_*d*_ *V*_*d*_). Signals appearing identically at both the *V*_+_ and *V*_−_ inputs at the same time will not be amplified. An example of an unwanted signal that could appear simultaneously at both amplifier inputs (i.e., a common-mode signal) would be electrical interference from power lines, which occurs at a frequency of 50 or 60 Hz, e.g., **Figure 10C**. Amplifiers also suppress EMG signals from distant muscles, which appear at the amplifier as common-mode signals. Ideally noise or interference signals would be completely rejected by the amplifier, however, in practice these unwanted common-mode voltages will receive some small amplification and the output voltage of the amplifier, *V*_*out*_, will contain some unwanted common-mode signals. A difference in the electrode impedance at the *V*_+_ and *V*_−_ inputs (due to inadequate skin preparation) can also increase the level of unwanted common-mode signals [see Equation 7 in Terminology Matrix in the CEDE project, (69) and https://www.robertomerletti.it/en/emg/material/tech/]. The ability of an amplifier to accurately reject common-mode signals (e.g., noise or interference) is quantified by the common-mode rejection ratio (CMRR), which is expressed in decibels^14^, Example (xiii) in Tutorial Code. When choosing an amplifier, the common-mode gain should be as small as possible and CMRR should be as large as possible (in practice the CMRR of the chosen amplifier should be >100 dB).

When setting the gain (*A*_*d*_) of an amplifier, care must be taken to ensure that the output voltage of the amplifier, *V*_*out*_, does not exceed the power supply voltage of the amplifier (e.g., ±10 V in Figure 7D). In the example shown in Figure 7D, the amplifier gain is 25, resulting in an output voltage that exceeds the power supply voltage. As a result, the output signal is clipped or limited at ± 10 V. Ideally, the gain of an amplifier should be as large as possible without exceeding the power supply voltage. Real amplifiers will also exhibit an “offset voltage” between the two terminals which results in an offset in the output voltage, see Figure 7E. This offset can be removed by high-pass filtering (see section Filtering) the EMG signal as typically takes place during the pre-amplification stage.

Finally, another important parameter to consider when choosing a bioelectric differential amplifier is the input impedance^15^. An internal input impedance, *Z*_*i*_, is present at each of the two input terminals (*V*_+_ and *V*_−_), typically consisting of a resistive component, *R*_*i*_, and a capacitive component, *C*_*i*_. The input impedance, *Z*_*i*_, acts as an obstacle to current flow between the input terminal and ground (reference), Figure 7B. An amplifier must have a high input impedance to optimally observe and record EMG activity without disturbing the voltage at the electrode. Amplifiers with high input impedance are also desired to minimize contamination from power line interference (see section Power Line Interference). Input impedance varies according to the frequency of the input signal and is therefore usually specified for a particular frequency. The amplifiers used in sEMG systems should have an input impedance >300 MΩ at 50 Hz (although minimum acceptable values will vary according to the type of electrode used). It should be noted that the choice of amplifier should be determined by the input impedance, *Z*_*i*_, and not the input resistance, *R*_*i*_, which is just one component of *Z*_*i*_.

### EMG Signal Sampling and Analog-to-Digital Conversion (A/D Conversion)

**Figure 8:**
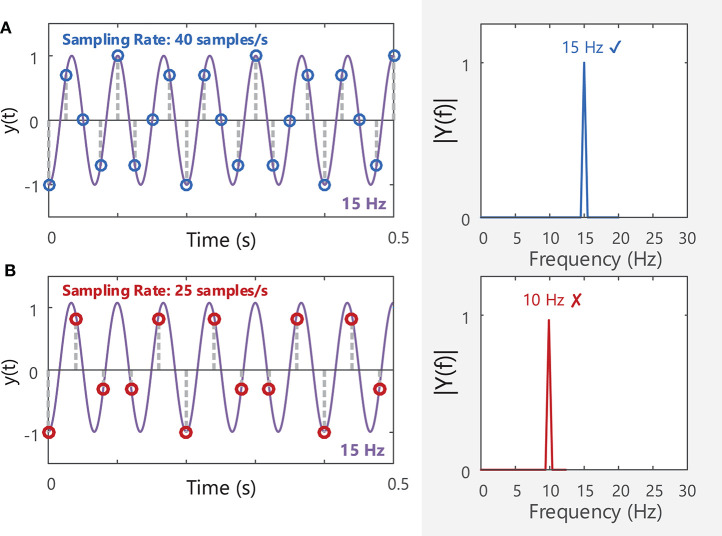
**(A)** A 15 Hz sine wave sampled at a rate of 40 samples/s will produce a corresponding peak at 15 Hz in the frequency domain (40 Hz is above the Nyquist frequency of 30 Hz). **(B)** The same sine wave sampled at 25 Hz (below the Nyquist frequency for the 15 Hz sine wave) will have a distorted amplitude spectrum, and the fundamental frequency of the signal is mis-identified as 10 Hz. See Example (iv) in Tutorial Code.

The final stage in recording EMG signals involves sampling the analog EMG signal so that it can be stored and processed as a digital signal. The EMG signal detected by the electrode is a continuous (analog) signal. In order to be stored and later processed, the analog signal must be first sampled to capture values at evenly spaced intervals or at a particular sampling frequency. To retain all the information contained within a signal, the signal must be sampled at a rate greater than twice the highest frequency component contained in the signal bandwidth (Nyquist's theorem^16^), Figure 8A. As the highest frequency component in the sEMG signal is ~450–500 Hz, sEMG signals are typically sampled at a minimum of 1,000 samples/s or Hz. If a lower sampling rate is chosen, the spectrum of the original signal may not be accurately represented, Figure 8B. Good practice specifies that signals should be recorded well-above this minimum sampling rate, for example at 2,000 Hz for typical sEMG signals. Before sampling the analog EMG signal, the signal should be low-pass filtered with an anti-aliasing filter^17^ with a cut-off frequency at or below half the desired sampling frequency. For example, if an EMG signal is to be sampled at 1,000 Hz, it should be low-pass filtered with a maximum cut-off frequency of 500 Hz. This attenuates/reduces frequency components >500 Hz, as these frequencies cannot be adequately represented by a sampling frequency of 1,000 Hz and would distort the EMG spectrum if not filtered out prior to sampling, **Figure 10B**. See section Filtering for further details on filtering and Nilsson et al. (70) for more information on the digital sampling of physiological signals.

Sampling converts the continuous analog signal into a discrete signal (a time series consisting of a sequence of distinct voltages). Each discrete voltage value can then be converted into a binary number consisting of a number of 1 and 0 s (or “bits”) that can be stored and further processed. Analog-to-digital signal conversion can introduce noise into the signal, as the true value of the analog signal will have to be rounded to the closest available discrete binary value at each sampling instance. These two values will never be exactly the same, and the small difference between them is termed the quantization error [for more details see Terminology Matrix in the CEDE project, (69)]. Choosing an analog-to-digital (A/D) converter with a higher number of bits will reduce quantization errors (A/D converters should typically have a resolution of at least 12 bits, Table 2), but will also increase the storage size of the output sEMG file.

**Table 2:** Summary of sEMG guidelines.

**Stage**	**Guidelines**
Choosing equipment (sEMG electrode, bioelectric amplifier, hardware filters, A/D converter)	• IED should be chosen based on the selectivity of the recording required (i.e., small IED when recording from muscles with a small surface area to avoid contamination from cross-talk) • Active or wireless electrodes may be preferred in experiments in which there is a high likelihood of motion artifact • Considerations for the choice of sEMG electrode are outlined in detail in Soderberg and Knutson (59) and Besomi et al. (60) • Hardware filters should typically band-pass filter the analog sEMG signal between 1 and 1,000 Hz (with a minimum sampling frequency of 5,000 Hz) (55, 70) • Bioelectric amplifiers with high input impedance are preferred [at least 300 MΩ at 50 Hz if small electrodes, e.g., 3 mm diameter, and floating amplifiers are used, and at least 80 MΩ at 50 Hz if small electrodes and battery-powered amplifiers are used, Merletti and Cerone (58)]. • An anti-aliasing filter (with cut-off frequency less than half the desired sampling rate) should be applied before A/D conversion • Choosing an analog-to-digital (A/D) converter with a higher number of bits can reduce quantization errors (the most commonly used A/D converters have resolutions of 12 or 16 bits)
Skin preparation	Skin should be exfoliated to remove dead skin cells, shaved if necessary and cleansed (see section skin preparation)
Electrode placement	Electrodes should be placed according to the SENIAM guidelines (http://seniam.org/sensor_location.htm), and are typically orientated along the direction of the muscle fibers and located on the midline of the muscle. The Atlas of Muscle Innervation Zones by Barbero et al. (6) provides quantitative evidence of the optimal placement of bipolar electrodes for 43 different muscles. An Atlas for Electrode Placement is also available in Criswell (4)
Noise and power line interference reduction	• Adequate skin preparation is one of the most important steps to reduce noise and interference in the sEMG signal • Power line interference can be reduced by moving power cables and equipment away from the subject, using wireless electrodes, shielding the electrode leads, keeping electrode leads short and/or twisting the leads together (minimize the closed loop area) and turning off fluorescent or LED lighting, section Power Line Interference
sEMG signal sampling	• Surface EMG signals must be sampled at a frequency >1,000 Hz (i.e., greater than twice the highest frequency component in the sEMG signal, typically around 500 Hz) If down-sampling sEMG signals to a lower sampling rate, the sEMG signal must be low-pass filtered with a cut-off frequency at or below half the desired sampling frequency to avoid aliasing or distortion of the signal, see section EMG signal sampling and analog-to-digital conversion (A/D conversion)
sEMG signal filtering (software)	• Surface EMG signals are typically band-pass filtered between 20 and 500 Hz with roll-off of 40 dB/decade, e.g., Figure 10C • Before down-sampling a sEMG signal, an anti-aliasing filter (with cut-off frequency less than half the desired sampling rate) should be applied to the signal • A notch filter centered at 50 or 60 Hz can be used to reduce power line interference when only an approximate estimate of EMG amplitude is required
sEMG Time/Frequency Domain Analysis	• sEMG signals are non-stationary and should be analyzed over short time epochs (0.5–1 s) when estimating the signal amplitude or the signal power spectrum during isometric contractions, see section EMG Signal Pre-processing and Analysis. Shorter time epochs should be used when analyzing dynamic contractions • sEMG signal amplitude is typically estimated using the root-mean-square (RMS) or the average rectified value (ARV) of the raw sEMG signal • Changes in the mean frequency or the median frequency of EMG power spectral density can be used to infer changes in MFCV (though are not a direct reflection of MFCV and are sensitive to other factors such as motor unit synchronization and recruitment)
sEMG signal normalization	Procedures for normalizing sEMG data are outlined in detail in Besomi et al. (22)
Reporting sEMG data	Standards for reporting EMG data are outlined in https://isek.org/wp-content/uploads/2015/05/Standards-for-Reporting-EMG-Data.pdf. Report the information requested in pages 103–105 of volume 8 of the SENIAM recommendations and in Merletti and Cerone (58)

### Noise in EMG Recordings

The choice of electrode and amplifier will determine the level of noise in the EMG signal. Random fluctuations in voltage can be observed in the voltage output from the amplifier, even when an electrode is placed on a fully relaxed muscle. This baseline noise arises from voltage fluctuations generated at the electrode-skin interface (see section Skin Preparation) and within the internal stages of the amplifier and its circuit components (the minimum baseline noise that can be achieved is typically >8 μV peak-to-peak). If the amplifier and analog to digital conversion meet recommended standards, the signal-to-noise ratio of an EMG signal will be primarily determined by the properties of the electrode-skin interface. Careful preparation of the skin surface prior to electrode placement is therefore essential to minimize baseline noise and optimize the signal-to-noise ratio in the sEMG signal [quality of contact can be reduced by over a factor of 10 with adequate skin preparation, (71)]. Sources of noise in sEMG signals are outlined in Table 3, and more detail on noise and artifacts in EMG signals can be found in Türker (77) and Merlo and Campanini (78).

**Table 3:** Sources of noise in the EMG signal.

**Noise source**	**Frequency range**	**Noise reduction**
Cable motion artifact	1–50 Hz	Ensure good contact between the electrode and skin. Use of short cables from the electrodes to the amplifier and securing these cables to minimize movement during the experiment. Use of the following electrode types: Ag-AgCl electrodes, wireless electrodes, and active electrodes^a^ (72). pavee High-pass filtering with a cut-off frequency between 10 and 20 Hz
Electrode motion artifact	<20 Hz	Minimize the electrode–skin impedance with appropriate skin preparation (i.e., skin abrasion) (73, 74) pavee High-pass filter the EMG signal with a cut-off frequency between 10 and 20 Hz [though higher cut-off frequencies may be more appropriate to filter sEMG recorded during dynamic movements (75)]
Electrode-skin Interface	<8 Hz, >1,000 Hz	Minimize the electrode–skin impedance. Choose a signal amplifier with a high input impedance
Power line interference	50 Hz in Europe and 60 Hz in North America and their harmonics (i.e., 100, 150, 200 Hz. etc., and 120, 180, 240 Hz, etc.)	Shield the EMG recording apparatus move it away from electrical equipment and power lines. Remove unnecessary electrical equipment nearby pavee Magnetically induced power line interference can be reduced by keeping the electrode leads short and/or by twisting the leads together, such that the loop area enclosed by the electrode leads, subject and signal amplifier is minimized pavee Experiments may be conducted within a Faraday cage, where available, to minimize electromagnetic interference
Electronic instrumentation	Typically, 10–500 Hz	Use of state-of-the-art recording systems
Quantization noise	Frequency independent (white noise)	Data acquisition system with sufficient A/D resolution [12-bit A/D convertor is typically regarded as the minimum acceptable for sEMG, with 16-bit or 32-bit A/D convertors preferred (76)]
Electrophysiological sources of noise (e.g., ECG artifact)	Typically, 0.1–100 Hz	Application of adaptive filtering in post-processing stage

a *An active electrode processes the EMG signal (filters and amplifies) within the electrode itself*.

#### Power Line Interference

One of the most common sources of unwanted fluctuations in voltage that contaminate or interfere with the detected sEMG signal is power line interference. Alternating currents (AC) in power lines and electrical wiring/equipment produce electromagnetic fields that fluctuate at the same frequency as the AC power supply (50 Hz in Europe and 60 Hz in the USA) and its harmonics (100, 150, 200 Hz in Europe and 120, 180, 240 Hz in the USA). These electromagnetic fields can induce currents in the electrode leads and the subject's body through parasitic capacitive coupling^18^ and electromagnetic induction^19^ (mostly in the closed loop formed by the electrode leads, the subject, and the amplifier). These currents produce an interference potential or voltage on the skin (which can reach an amplitude of several volts) that is detected by the recording electrode, appearing as a common-mode input to the differential amplifier.

The magnitude of this power line artifact can be reduced by minimizing any difference in the electrode-skin impedance between the two amplifier input terminals through adequate skin preparation, and by choosing an amplifier with a high input impedance. If the electrode-skin impedances at the two terminals (*Z*_*e*1_and *Z*_*e*2_, respectively) are unequal, a portion of the unwanted common-mode interference signal will differ between *V*_+_ and *V*_−_. This difference will be amplified by the differential gain *A*_*d*_ of the amplifier^20^.

Other methods used to reduce power line artifact include moving power cables and equipment away from the subject, using wireless electrodes, shielding the electrode leads, keeping electrode leads short, and/or twisting the leads together (minimize the closed loop area), turning off fluorescent or LED lighting, and using the driven-right leg technique^21^.

## EMG Signal Pre-Processing and Analysis

The sEMG signal can be used to infer information about the behavior of the underlying motor unit population. Useful information can be obtained from the time-domain EMG signal, and by examining the power spectrum of the sEMG signal in the frequency domain, Figures 5D,E. In the time domain, the amplitude of the sEMG signal can be used to determine whether a muscle is activated, i.e., “on” or “off.” An increase in the amplitude of the sEMG signal can also indicate that additional motor units are being recruited or motor units are discharging faster to increase force production. In the frequency domain, alterations in the amplitude, or power spectrum of the sEMG can provide insights into changes in muscle fiber conduction velocity^22^ and are often used in the assessment of muscle fatigue (79). Standards for reporting EMG data are outlined in Merletti (80) (https://isek.org/resources/).

### Power Spectral Density Estimation

**Figure 9:**
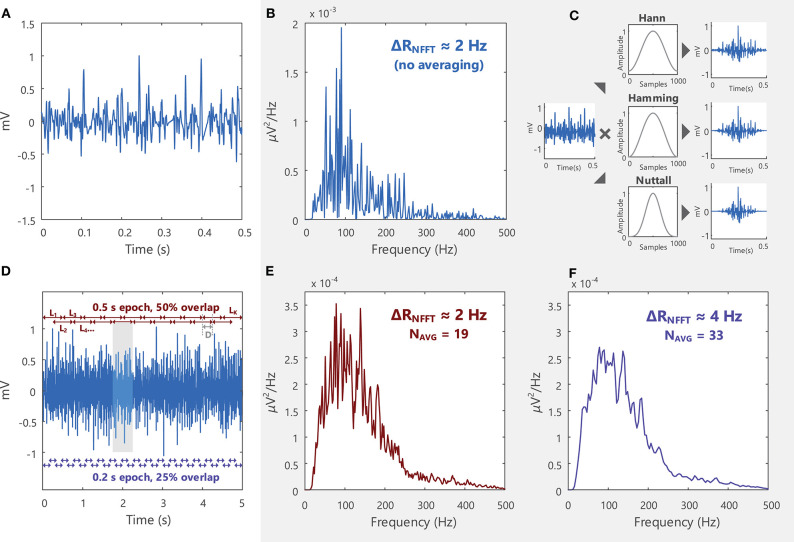
**(A)** An EMG signal sampled at 2,000 samples/s in the time domain and **(B)** the power spectrum of the signal in the frequency domain. The signal spectrum contains several spurious peaks. **(C)** Welch's method breaks the total signal (5 s long, shown in **D**) into shorter segments (0.5 s) and multiplies (convolves) each segment by a window function (some examples are the Hann, Hamming, and Nuttall windows) before averaging all the modified segments. See Example (viii) in Tutorial Code. **(D)** In Welch's averaging method, the EMG signal is divided into a number of segments (K). K depends on the length of the segment (L) and the degree of overlap between successive segments, Equation 3 in Supplementary Material. Each successive segment starts D samples after the previous segments. **(E)** By obtaining an average power spectral density across K segments, the spurious peaks in **(B)** are reduced. **(F)** The smoothness of the power spectral density function can be increased by increasing K (i.e., increasing the number of averages, N_AVG_), which can be achieved by decreasing the length of L or increasing the overlap between segments. See Example (vii) in Tutorial Code.

The frequency content of an EMG signal can be examined by applying the Fourier Transform, as described in section Frequency Domain Analysis. EMG signals are typically analyzed over short time intervals or epochs (0.5–1 s) in the time and frequency domains, as they are generated by non-stationary processes^23^. In the frequency domain, these short duration signals have a noisy power spectrum, Figure 9B. To obtain a smoother power spectrum representation of the EMG signal, the total signal length can be divided into short segments or epochs containing a fixed number of samples, *L*, which can overlap in time. The power spectral density can then be estimated for each signal epoch, and these local estimates averaged to obtain the power spectral density of the entire signal length, see Supplementary Material: Advanced Topics, section A.1 and Figure 9 for more details. A “smoother” power spectral density estimate can be obtained by decreasing the length of *L* or increasing the overlap between successive signal segments, Figures 9E,F.

### Filtering

**Figure 10:**
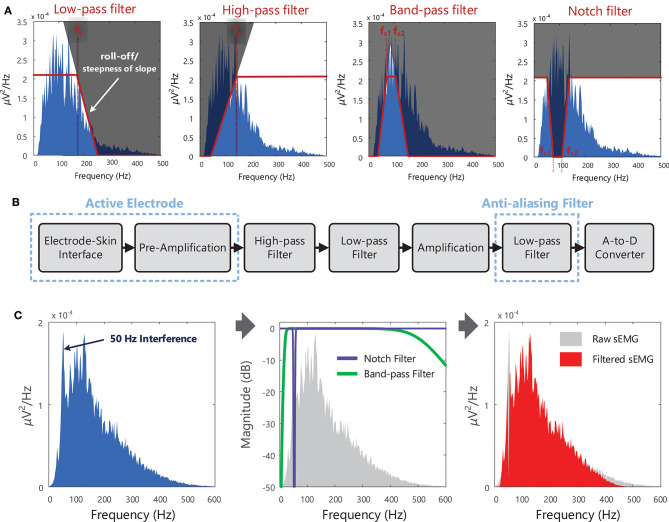
**(A)** Different types of filters that can be used to shape the EMG spectrum, to keep, remove, or attenuate certain frequency components of the EMG signal + noise. In the example shown, the low-pass filter has a cut-off frequency (f_c_) of 170 Hz and the high-pass filter has a cut-off frequency of 140 Hz. The band-pass and notch filters have lower cut-off frequencies (f_c1_) of 70 Hz and upper cut-off frequencies (f_c2_) of 108 Hz. **(B)** Schematic of the typical stages in recording surface EMG signals, with filtering at several points along the process (filters that operate on the analog signal, i.e., hardware filters). With active electrodes, pre-amplification is performed within the electrode itself (rather than the amplification being performed in an external circuit), see section Choice of Amplifier. EMG signals are low-pass filtered before sampling to suppress high-frequency components and prevent the distortion of the spectral content, see Figure 8. See Examples (ix) and (x) in Tutorial Code. **(C)** An example of a noisy sEMG signal contaminated with 50 Hz interference, the frequency response of a 50 Hz notch filter and a 20–500 Hz band-pass filter (the figure indicates how much the sEMG signal is attenuated by the filter, in dB, at each frequency), and filtered sEMG spectrum (after notch and band-pass filtering the raw sEMG signal). Note that notch filters are typically used when only an approximate estimate of EMG amplitude is required.

The quality of a recorded EMG signal is influenced by the signal-to-noise ratio, which describes the relative power of the “true” EMG signal to that of unwanted or artifactual signal components (noise, interference etc.) in the overall signal. Methods for reducing noise contamination in the EMG signal are outlined in Section(s) Choice of Electrode, Choice of Amplifier, and Noise in EMG Recordings, and in Clancy et al. (76). However, even with well-designed instrumentation and careful skin preparation, there will be some noise and/or interference (i.e., unwanted signals) present in the EMG signal detected from the skin surface. Although noise arising from the electronic circuitry is present across a broad frequency range (from 0 Hz to several thousand Hz), electrical signals from other noise sources can have most of their energy contained within specific frequency bands. For example, most of the power in electrical signals occurring due to motion artifact^24^ will lie below 20 Hz, Table 3. Different types of filters [low-pass, high-pass, band-pass, and notch filters, for definitions on each filter type see the Terminology Matrix in the CEDE project (69)] can be used to shape the EMG power spectrum and to remove or attenuate frequency components that are likely due to noise, Figure 10 and Example (x) in Tutorial Code. The signal-to-noise ratio in the detected EMG signal can be improved using hardware, Figure 10B. Software filters and other signal processing techniques can also be applied to the recorded EMG signal to further attenuate (i.e., reduce) unwanted frequency components.

Surface EMG signals are typically band-pass filtered between 20 and 500 Hz (with roll-off of 40 dB/decade or 12 dB/oct ^25^, see Figure 10A) to remove the electrical noise at frequencies below a cut-off frequency of 20 Hz and above a cut-off frequency of 500 Hz. Power line interference can be reduced with a notch filter centered at 50 or 60 Hz, see Figure 10A. However, this approach removes both wanted and unwanted signal components, and is thus only recommended when an approximate estimate of EMG amplitude is required. For other EMG applications, more advanced adaptive filtering methods may be necessary to remove interference and preserve spectral content, for example to remove electrocardiographic signal (ECG, the electrical activity of the heart) artifacts from recordings from back or diaphragm muscles (81). If the sampling rate of the EMG signal is to be reduced before further processing (i.e., down-sampled), the signal should be low-pass filtered with a cut-off frequency at or below half of the new lower sampling frequency. This is a necessary step in order to suppress high-frequency signal components and prevent aliasing or distortion of the signal, see section EMG Signal Sampling and Analog-to-Digital Conversion (A/D Conversion). Further information on filtering physiological signals can be found in MacCabee and Hassan (82).

### Surface EMG Amplitude Features (Time Domain)

**Figure 11:**
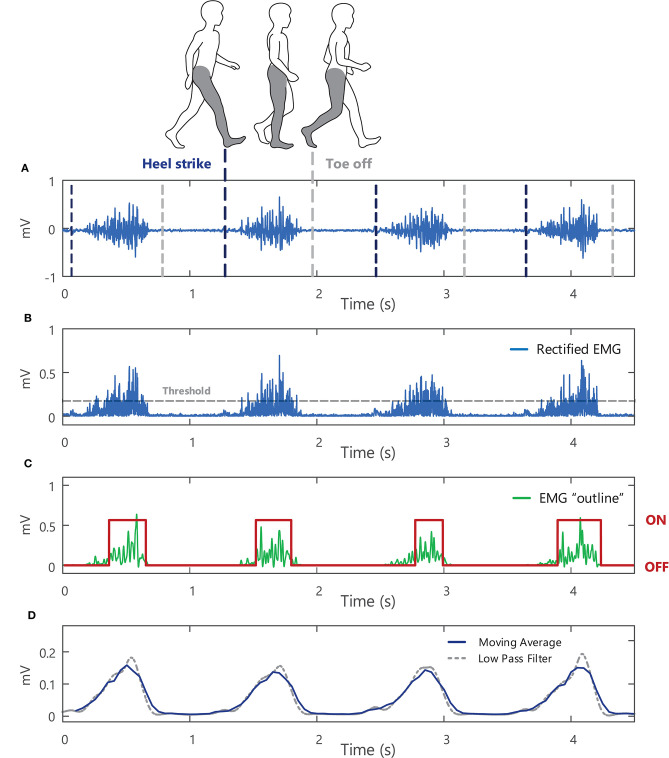
**(A)** A raw surface EMG signal recorded from the soleus muscle during walking, with vertical lines to indicate where heel strike and toe off occur during the gait cycle. **(B)** The absolute value or rectified surface EMG signal. **(C)** The outline or “shape” of the rectified EMG signal obtained by low-pass filtering the EMG signal at 50 Hz (after applying the Teager-Kaiser Energy Operator to the signal) with muscle onset times shown in red. **(D)** A 0.2-s moving average of the rectified surface EMG signal (with 25% overlap) and an average obtained using a 5-Hz low-pass filter (with the filter applied twice so that there is no time delay in the filter output, see Example (xiv) in Tutorial Code).

The amplitude of an EMG signal varies randomly above and below 0 V, thus there is no information gained from the average of the raw EMG signal (i.e., the mean is zero^26^). To quantify the amplitude of a sEMG signal, a transformation or function must be applied to the raw EMG signal, Figure 11A. The two most common functions are the root mean square value (RMS) and the average rectified value (ARV) (or mean absolute value, MAV) of the EMG signal amplitude (see Example (xvi) in Tutorial Code). The RMS of the EMG signal is an estimate of the standard deviation of the signal, i.e., a measure of how much the signal differs from zero (for an EMG signal with a mean of 0 V). It is equal to the square root of the total power contained within the EMG signal. The ARV of the EMG signal calculates the mean of the rectified or absolute value^27^ of the EMG signal amplitude, Figure 11B. The ARV is proportional to RMS of the EMG amplitude when the level of muscle activity is sufficiently high (83).

The sEMG signal amplitude is typically calculated over short time intervals or windows during which the signal can be assumed to be approximately stationary. For isometric contractions this corresponds to windows or epochs of ~0.5–1 s, with shorter duration epochs typically used to capture amplitude changes during dynamic contractions. Using a moving average window, EMG amplitude is estimated for a short section (or window) of the EMG signal. The EMG window under analysis is then shifted forward in time, incrementally, to obtain an estimate for each sequential section of the signal. The moving average is a simple method to smooth sEMG data, acting as a low pass filter and reducing random fluctuations, Figure 11D. Short duration windows or epochs allow rapid changes in muscle activity to be detected, whereas longer epochs produce a stronger smoothing effect. Calculating the moving average with epochs that overlap in time (50% overlap or more between successive windows) can further reduce the overall variability of the EMG amplitude profile but can reduce the ability to detect sudden changes in signal amplitude. Alternatively, the rectified EMG signal can be low pass filtered to obtain the “shape” and outline changes in the amplitude of the EMG signal, Figure 11C. Lower cut-off frequencies will result in a smoother EMG amplitude profile (e.g., the 5 Hz low-pass filter in Figure 11D results in a smoother signal than the output obtained with a 50 Hz low-pass filter, Figure 11B).

An increase in the amplitude of the sEMG signal can indicate an increase in muscle activation (provided there is no cross-talk from other muscles, see section Choice of Electrode), with additional motor units recruited to increase force production and/or a change in motor unit firing rates. In Example (xv) in Tutorial Code, the sEMG signal is shown at three different levels of voluntary muscle contraction force, exhibiting an increase in the RMS/ARV of the EMG signal amplitude as the level of muscle force is increased. Both the amplitude and frequency characteristics of the raw EMG signal are sensitive to many factors, some of which can be experimentally controlled (extrinsic factors, e.g., electrode type and orientation/location, see section Choice of Electrode), and others which typically cannot be controlled (intrinsic factors) and depend on the physiological, anatomical, and biochemical characteristics of the muscle under investigation (e.g., muscle fiber length/cross-sectional area/orientation/composition, level of subcutaneous fat, number of motor units). de Luca (39) provides a comprehensive description of the different factors influencing the sEMG, covering both causative factors (those that determine the basic composition of the EMG signal detected) and deterministic factors (those that directly influence the information content of the EMG signal). EMG signals recorded under different conditions (different subjects/muscles/measurement sessions/electrode positions) are thus essentially measured on different scales. For example, the RMS amplitude of the sEMG signal recorded during a given measurement session could be less than that recorded from the same subject, force level, and task on a different day [or on the same day due to changes in electrode position, in temperature, Winkel and Jørgensen (84), or in the electrode tissue interface]. To enable comparisons between different recording conditions and subjects, the sEMG amplitude must typically be normalized^28^ to a reference value^29^, which converts the raw EMG signal from volts (absolute scale) to a percentage of the reference value (relative scale) (85), see also Besomi et al. (22). This reference value is often chosen as the EMG amplitude recorded during a maximal voluntary contraction in the muscle of interest for each subject (i.e., 100% MVC). However, other signal normalization techniques may be more appropriate for certain subject groups, muscles, or experimental protocols (for example, maximal effort contractions may not be possible for older subjects or patient groups) (85, 86). Normalization to the EMG amplitude during submaximal contractions or to the M-wave amplitude^30^ are other commonly used reference values.

A moving average of the normalized, rectified EMG signal amplitude can be used to determine whether a particular muscle is activated during a task. One method for establishing whether a muscle is “active” or “inactive” involves setting a threshold for muscle activation, e.g., Figure 11B. The threshold is typically chosen as a percentage of the mean RMS amplitude of the EMG signal (other estimates of the signal standard deviation can also be used), and when the RMS of the EMG signal goes above this threshold, the muscle is “on.” Transformations can be applied to the EMG signal to improve the accuracy in the muscle activation onset timing. In Example (xiv) in Tutorial Code the Teager-Kaiser Energy Operator transformation is applied to the EMG signal and a threshold is set for muscle activation (87) [the Teager-Kaiser Energy Operator can also be applied to accelerometry data (88, 89)]. When the normalized EMG signal is greater than this threshold, the muscle is considered active or “on,” Figure 11C.

### Surface EMG Spectral Features (Frequency Domain)

**Figure 12:**
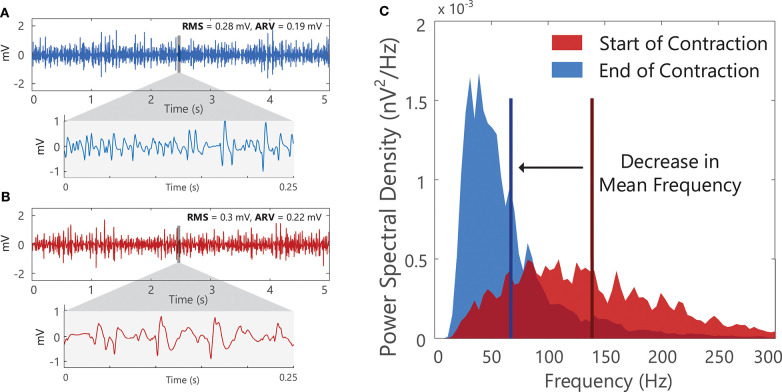
A 5-s segment of surface EMG signal from at **(A)** the start and **(B)** the end of a fatiguing isometric contraction in the first dorsal interosseous muscle. With fatigue the duration of the motor unit action potential lengthens, and there is a shift in the surface EMG signal to lower frequencies. **(C)** The shape of the power spectral density of the surface EMG segments shown in **(A)** and **(B)**. A decrease in the mean frequency (vertical line) of the surface EMG signal is observed, accompanied by an increase in the surface EMG amplitude, see increase in RMS and ARV of the surface EMG amplitude in **(B)**. See Example (xvii) in Tutorial Code.

Examining sEMG signals in the frequency domain can provide information on changes in the frequency content of the signal, manifesting as alterations in the shape of the EMG amplitude/power spectrum, Figure 12C. Changes in the mean frequency or median frequency of EMG power spectral density are often used to track peripheral muscle fatigue, see Example (xvii) in Tutorial Code. During fatiguing muscle contractions there are a number of ionic and metabolic changes within the muscle that slow muscle fiber conduction velocity. As muscle fiber conduction velocity decreases (i.e., as the speed at which action potentials travel along the muscle fibers reduces), the action potentials recorded by the electrode will appear longer in duration and the action potential will have a lower frequency content, Figure 1D. This results in a compression of the sEMG power spectrum toward lower frequencies with a consequential reduction in the mean/median frequency of the sEMG signal, Figure 12. During fatiguing isometric muscle contractions, the decrease in mean/median frequency is typically accompanied by an increase in the sEMG amplitude (see increase in RMS and ARV of the sEMG amplitude in Figure 12B). The changes in the amplitude and spectral parameters of the sEMG during sustained muscle contractions are often referred to as “myoelectric manifestations of fatigue.” The mean and median frequency of the sEMG power spectral density are also sensitive to motor unit synchronization^31^, which increases during fatiguing contractions (90). Changes in skin and muscle temperature also influence the EMG power spectrum and median frequency as muscle fiber conduction velocity decreases with temperature reduction (84, 91). More advanced time-frequency transforms, such as the wavelet transform^32^, can be applied to investigate non-stationary sEMG signals that exhibit rapid temporal variations in frequency content (i.e., during dynamic muscle contractions).

## Surface EMG Limitations

The primary advantage of sEMG over intramuscular EMG, along with its non-invasive nature, is that the signals are relatively straightforward to record and analyze (with some basic signal processing knowledge). It also provides an estimate of the overall activity of a muscle or group of muscles in contrast to the more selective nature of intramuscular recordings. Surface EMG, however, is only suitable for recording from superficial muscles and is not appropriate for recording deep muscle activity. Recording from small muscles without contamination from surrounding muscles can be difficult and signals can be prone to cross-talk, particularly over regions where there is substantial subcutaneous fat (66, 92). Furthermore, it should be emphasized that the sEMG signal is not a direct measure of the behavior or properties of the underlying motor unit population. The properties of the sEMG signal are determined by the number of action potentials generated by active motor units within the detection volume of the sEMG electrodes in addition to the shape of these action potential waveforms. It is an interference signal, comprised of the superposition of many action potentials leading to constructive and destructive interference, and is sensitive to many other factors (e.g., the recording instrumentation, crosstalk from other muscles, external noise/interference, see section Surface EMG Amplitude Features (Time Domain) for further details). Unlike signals recorded using surface EMG grids (section Practical Applications of Surface EMG in the Clinic), conventional bipolar sEMG cannot provide direct information on individual motor units. The amplitude and frequency characteristics of the sEMG signal can thus only be used to infer changes in motor unit activity and muscle fiber conduction velocity, respectively. It should also be emphasized that due to the variability of sEMG measurements, sEMG signals recorded under different conditions (different subjects/muscles/measurement sessions/electrode positions) can only be reliably compared after applying correct normalization procedure [see Besomi et al. (22) for details].

## Discussion

Sensor technology has developed rapidly over the past 30 years, but physiotherapy education has lagged behind in training new therapists to use the newest sEMG innovations in clinical practice. For sEMG to be widely adopted as a quantitative assessment tool, therapists need to see how this technology can directly benefit everyday practice, with guidance from a trusted source such as an educator or clinical mentor. Therapists also need to be empowered with the technical knowledge to enable them to record and analyze their own data. Through education this can be provided in a succinct and accessible manner that removes the perceived complexity of the technology. Modern curricula for physiotherapists may provide a basic introduction to recording and analyzing sEMG signals. However, without practical experience working with sEMG signals, and a comprehensive overview of the technical aspects involved in recording and analyzing the signal, it is difficult to bridge the gap between knowledge of the theory and the ability to apply this in practice. This paper provides a concise guide that simplifies and condenses the relevant information for recording and processing sEMG. Although this material and accompanying tutorial may help to remove some of the educational barriers (i.e., the perceived difficulty and relevance of sEMG as a clinical tool), access to information alone is not enough to motivate therapists to adopt sEMG in their own practice. To effectively promote and encourage the use of sEMG in clinic, practical experience working with sEMG needs to be embedded into education in the form of workshops, tutorials, or course placements. A deeper understanding of signal processing concepts, and the confidence to apply these methods to sEMG signals is hard to achieve without first-hand experience of working with sEMG signals in a guided setting. Placements in clinics or research labs that use sEMG can also expose students to the utility of sEMG and how it is used by experienced practitioners demonstrating the practical benefits. This can be facilitated by establishing links between local clinics and physiotherapy and biomedical engineering departments within universities. Tutorials that form part of the biomedical engineering course could be adapted for a clinical audience and offered to physiotherapy students. Prior experience either using sEMG themselves or observing first-hand how it can be successfully implemented in clinic is likely to be a deciding factor in a practitioner's decision to adopt sEMG in their own practice.

A lack of prior exposure to sEMG signal analysis may thus present the greatest barrier to physiotherapists wishing to incorporate sEMG as a measurement tool in their clinical practice. However, therapists may also be discouraged by the fact that many resources and scientific papers currently available for sEMG analysis are targeted at a technical audience. These resources often assume the reader has prior coding experience and the resources to develop customized code for signal analysis. Clinicians and therapists may alternatively opt to use software packages to extract relevant features from the sEMG signal where the underlying calculations may not be evident. Even in these cases, a basic understanding of signal processing is still important so that the user can select appropriate analysis parameters for different conditions and justify this choice when interpreting and reporting their results. To encourage the uptake of sEMG in clinic, this paper presents an overview of key topics that could be used to guide the content of lectures/tutorials on sEMG in the curricula for physiotherapists or used to form the base of an elective module on EMG applications. It is important to note that some of the simplest applications of sEMG (that require minimal knowledge of sEMG concepts and are relatively easy to implement and interpret) may be the most useful in practice (e.g., visual feedback on muscle activation). The basics of sEMG could be relayed in a single workshop/practical (~ 3 h), which could be incorporated into the undergraduate curriculum for physiotherapists. More advanced signal analysis could be then be covered in postgraduate or elective modules. Combining sEMG theory with practical classes in basic computer programming (in addition to providing code and lectures online to support the material covered) is an effective way to teach sEMG concepts to physiotherapists, break down its perceived complexity, and encourage them to incorporate sEMG as a measurement tool in their practice (93).

Sample sEMG signals are provided in the Supplementary Material (and at https://doi.org/10.5281/zenodo.4001609) accompanying this paper that could be used in sEMG tutorials in cases where it is not feasible to record sEMG signals. Sample codes for signal analysis are also provided, illustrating how to extract commonly used features such as the RMS or ARV amplitude and the mean or median frequency of sEMG (see Key Functions Code). The background material and the signal analysis code are intended to be used in parallel, often practical examples can help to simplify more difficult concepts. The examples also illustrate the importance of understanding these signal processing concepts, by illustrating how they can influence outcome measures. The parameters used to analyze the sEMG signals in these examples can also be altered in the code to directly examine the effect on signal output, which can provide a greater insight into the function and relevance of each signal parameter. Though the material is designed to be accessible, it will require a time investment to read, run the accompanying code and understand the output. However, for those interested in incorporating sEMG into their practice, time dedicated to developing a deeper understanding of the technical aspects of sEMG will be rewarded as it will enable them to optimize and tailor their recording and analysis to address the problems they are most interested in. While some may wish to leave sEMG recording and processing to clinical engineers or technicians, with an understanding of the topics outlined in this paper and some investment of time, there is no reason that recording and processing cannot be performed within the clinic by physiotherapists themselves. Therapists themselves are the ones best placed to know where sEMG could be most useful and practical in clinic, and what is practical to implement during the time allotted for a patient appointment. Even where rehabilitation engineering support and resources are available, a common language and understanding enhances the collaboration between engineers and therapists to ensure the most appropriate application of sEMG to address each research question.

More widespread use of sEMG in clinical practice should contribute to increasing the reliability and reproducibility of studies evaluating the efficacy of healthcare interventions in physical and rehabilitative medicine, however, the full potential of sEMG in clinical assessment and neurorehabilitation has yet to be realized. Through the material presented here we aim to facilitate this by addressing some of the educational and technical barriers that limit the clinical translation of sEMG.

## Data Availability Statement

The datasets presented in this study can be found in online repositories. The names of the repository/repositories and accession number(s) can be found in https://doi.org/10.5281/zenodo.4001609.

## Author Contributions

LM and ML conceived and designed research and drafted manuscript. LM prepared figures. LM, GD, and ML edited and revised manuscript and approved final version of manuscript. All authors contributed to the article and approved the submitted version.

## Conflict of Interest

The authors declare that the research was conducted in the absence of any commercial or financial relationships that could be construed as a potential conflict of interest.

## Footnotes

^1^Sensor made of conductive material. The individual contacts within an electrode sensor can also be termed “electrodes” (but are here referred to as “electrode contacts”).

^2^It is important to note that measuring the electrical activity of the muscle with sEMG is not equivalent to measuring the tension produced within the muscle, as the EMG signal precedes mechanical muscle activity. It is also possible for electrical and mechanical muscle activity to occur independently from one another.

^3^A motor unit is the smallest functional unit of a muscle, it consists of a motoneuron and the group of muscle fibers it innervates.

^4^A muscle contraction in which muscle tension is produced with no change in muscle length. It is often assumed that this is the case when there is no change in joint or limb position.

^5^Central fatigue encompasses decreases in descending motor commands from the brain to spinal motoneurons, reduced excitatory afferent input, and decreases in motoneuron responsiveness.

^6^Peripheral fatigue refers to changes occurring beyond the motoneuron, including changes within the muscle fibers.

^7^A CMAP is generated when the motor nerve supplying the muscle is electrically stimulated, causing the muscle to contract. It is the summated electrical response of the activated motor units within the muscle.

^8^The Fourier Transform is a mathematical function or technique that enables a signal to be separated and represented in terms of sine (or cosine) waves of different frequencies (which sum to reconstruct the original signal).

^9^The term power spectrum and power spectral density are often used interchangeably. To obtain the power spectral density (measured in V^2^/Hz), the power spectrum is normalized by dividing by the frequency resolution (i.e., in the case of a 1 Hz frequency resolution, the magnitude of the power spectrum remains the same).

^10^Too much conductive gel will allow current to flow along the gel, directly connecting the electrode contacts to each other, i.e., short-circuiting.

^11^Resistance to the flow of the electrical current at the contact point between the metal electrode and the skin surface.

^12^An ion is an atom or group of atoms with an electric charge. Ions (e.g., sodium, chloride, potassium, calcium, and magnesium ions) enable the flow of electrical signals through the body.

^13^A region of a muscle where there is a concentration in the distribution of neuromuscular junctions (i.e., synaptic connections between an axon terminal of a motoneuron and a skeletal muscle fiber).

^14^A unit used to express the ratio or relative magnitudes of two electrical signals on a logarithmic scale.

^15^A measure of the opposition to the flow of current into each input terminal of an amplifier, as a function of frequency.

^16^The Nyquist theorem states that an analog signal can be converted to a digital signal and reconstructed without error if the sampling rate is greater than twice the highest frequency component in the analog signal.

^17^Aliasing is the distortion of amplitude/power spectrum of a signal when the signal is sampled at a rate that is too low to accurately reconstruct the original signal.

^18^The unwanted transfer of electrical current from one current-carrying conductor (e.g., electrical wiring) to another conductor that is physically close (e.g., the human body), due to the interaction of the electric fields surrounding the conductors.

^19^The production of a current in a conductor that arises due to the voltage produced by the changing magnetic field from another nearby current-carrying conductor.

^20^Minimizing the difference between *Z*_*e*1_ and *Z*_*e*2_ and maximizing the amplifier input impedance, *Z*_*i*_, reduces the power line interference: $V_{interference}=\frac{A_{d}V_{CM}(Z_{e1}-Z_{e2})}{Z_{i}}.$.

^21^A method whereby the common-mode voltage (due to power line interference) on the body is negatively fed back to a third electrode, which is placed on the body to “cancel out” or reduce the power line interference.

^22^The speed at which an action potential propagates or travels along a muscle fiber.

^23^EMG signals exhibit non-stationarity as the statistical properties of the processes that generate the signal will change over time. However, it is often assumed that the processes generating the EMG signal are stationary over short time intervals (i.e., it exhibits quasi-stationarity).

^24^Unwanted voltage fluctuations present in the surface EMG signal due to movement between the electrode and the underlying skin or the movement of cables connected to the recording electrode.

^25^Filter roll-off describes the steepness of the transition between frequencies that pass through the filter unattenuated (passband) and frequencies that are removed by the filter (stopband), see Figure 10A. It is measured in either decibels/decade or decibels/octave, where a decade is a 10-fold increase in frequency and an octave is a 2-fold increase in frequency.

^26^If the mean value of the EMG signal is non-zero, the mean should be subtracted from the signal before any further processing, as it is an artefact introduced by the recording instrumentation and electronics (see voltage offset).

^27^All negative (minus) voltage value become positive.

^28^EMG signal normalization re-scales the EMG signal amplitude by dividing the signal by a reference EMG amplitude.

^29^A reference value should be obtained under standardized and reproducible conditions and have good test-retest repeatability.

^30^An M-wave is the summated electrical response of motor units within a muscle, evoked by electrically stimulating the muscle's motor nerve.

^31^An increased tendency for two or more motor units to discharge together or within a few milliseconds of one another.

^32^A transform that deconstructs a time domain signal into a sum of “wavelets” or short waveforms of different scales and time shifts, to produce a time-frequency representation of a time domain signal.

## Supplementary Material

Supplementary Data Sheet 1 Further reading.

Supplementary Data Sheet 2 Appendix: Advanced topics.

Supplementary Data Sheet 3 Tutorial codes.
