# Supplementary material for: Analysis and Biophysics of Surface EMG for Physiotherapists and Kinesiologists: Toward a Common Language With Rehabilitation Engineers
Source: Front Neurol. 2020 Oct 15;11:576729. doi: 10.3389/fneur.2020.576729 (PMC7594523; doi:10.3389/fneur.2020.576729)
Supplement: Supplementary Data Sheet 1 — Further reading. [file Data_Sheet_1.DOCX]

# Further Reading

Sample surface EMG signals and analysis codes are provided in the supplementary material accompanying this paper - https://doi.org/10.5281/zenodo.4001609

**Physiology**

***Books***

Kernell, D. (2006). *The motoneurone and its muscle fibres.*

Purves, D., Augustine, G. J., Fitzpatrick, D., Hall, W. C., LaMantia, A. S., McNamara, J. O., & White, L. E. (2004). *Neuroscience*, Sinauer Associates. *Inc., USA*. **See Chapter 15**: Lower Motor Neuron Circuits and Motor Control

Windhorst, U., & Johansson, H. (Eds.). (2012) *Modern techniques in neuroscience research.* Springer Science & Business Media.

Pfaff, D. W., & Volkow, N. D. (Eds.). (2016). *Neuroscience in the 21st century: from basic to clinical*. Springer.

Akay, M. (2006). *Wiley encyclopedia of biomedical engineering*. (in particular, the sections “Electromyography (EMG), Electrodes and Equipment for” and “Motor Unit”)

Cope, T. C. (2001). *Motor neurobiology of the spinal cord*. CRC Press.

Binder, M. D., Heckman, C. J., & Powers, R. K. (2010). The physiological control of motoneuron activity. *Comprehensive physiology*, 3-53.

**EMG**

***Books (aimed at clinicians)***

Barbero, M., Merletti, R., & Rainoldi, A. (2012). *Atlas of muscle innervation zones: understanding surface electromyography and its applications*. Springer Science & Business Media.

Basmajian, J. V., & De Luca, C. J. (1985). *Description and analysis of the EMG signal. Muscles alive: their functions revealed by electromyography.*

Kamen, G., & Gabriel, D. A. (2010). *Essentials of Electromyography.*

Criswell, E. (2010). *Cram's introduction to surface electromyography.* Jones & Bartlett Publishers.

Loeb, G. E., Loeb, G., & Gans, C. (1986). *Electromyography for experimentalists.* University of Chicago Press.

Perotto, A. O. (2011). Anatomical guide for the electromyographer: the limbs and trunk. Charles C Thomas Publisher.

Robertson, G. E., Caldwell, G. E., Hamill, J., Kamen, G., & Whittlesey, S. (2013). *Research methods in biomechanics*. Human kinetics. **See Chapter 8**: Electromyographic Kinesiology

Schwartz, M. S., & Andrasik, F. (Eds.). (2017). *Biofeedback: A practitioner's guide*. Guilford Publications.

Schwartz M. *EMG Methods for Evaluating Muscle and Nerve Function.* London, UK: Intech Open (2012).

Steele C. *Applications of EMG in Clinical and Sport Medicine.* London, UK: Intech Open (2012).

***Books (general)***

Merletti, R., & Farina, D. (Eds.). (2016). *Surface electromyography: physiology, engineering, and applications. John Wiley & Sons.*

Pierce, P. A. (2013). *Fatigue: neural and muscular mechanisms* (Vol. 384). Springer Science & Business Media.

Enoka, R. M. (2008). *Neuromechanics of human movement*. Human kinetics.

Winters, J. M., & Crago, P. E. (Eds.). (2012). *Biomechanics and neural control of posture and movement*. Springer Science & Business Media.Perry, J., & Davids, J. R. (1992). Gait analysis: normal and pathological function. *Journal of Pediatric Orthopaedics*, *12*(6), 815.

***EMG: Guides for Clinicians***

Kamen, G., & Caldwell, G. E. (1996). Physiology and interpretation of the electromyogram. *Journal of Clinical Neurophysiology*, *13*(5), 366-384.

Soderberg, G. L., & Knutson, L. M. (2000). A guide for use and interpretation of kinesiologic electromyographic data. *Physical therapy*, *80*(5), 485-498.

Hogrel, J. Y. (2005). Clinical applications of surface electromyography in neuromuscular disorders. *Neurophysiologie Clinique/Clinical Neurophysiology*, *35*(2-3), 59-71.Merlo, A., & Campanini, I. (2010). Technical aspects of surface electromyography for clinicians. *The open rehabilitation journal*, *3*(1).

Besomi, M., Hodges, P. W., Clancy, E. A., van Dieën, J., Hug, F., Lowery, M., ... & Carson, R. G. (2020). Consensus for experimental design in electromyography (CEDE) project: Amplitude normalization matrix. *Journal of Electromyography and Kinesiology*, 102438.

***Websites/Online Resources***

SENIAM guidelines (<http://seniam.org/sensor_location.htm>)

Merletti (2016). Movement control and non-invasive electromyography: Project CoMES. (<https://www.robertomerletti.it/en/emg/material/teaching/>) – section targeted at clinicians.

Konrad, P. (2005). The ABC of EMG. *A practical introduction to kinesiological electromyography*, *1*(2005), 30-5. [http://www.noraxon.com/](http://resourcelists.ntu.ac.uk/link?url=http%3A%2F%2Fwww.noraxon.com%2F&sig=94d8292cefab42b97905042460aa2ac63bb80e09390c7a7b046903213fbbfbeb)

Day (2002). Important Factors in Surface EMG Measurement (<http://www.bortec.ca/pages/resources.htm>)

De Luca, G. (2003). Fundamental concepts in EMG signal acquisition.

**Signal Processing Theory**

***Books***

Buck, J. R., Oppenheim, A. V., & Schafer, R. W. (1999). Discrete-time signal processing. ed. Prentice Hall.

Hsu, H. P., & Hsu, H. P. (2014). *Signals and systems* (Vol. 8). New York: McGraw-Hill Education.

***Basic Concepts of Electronics and Signal Processing: Guides for Clinicians***

Nilsson, J., Panizza, M., and Hallett, M. (1993). Principles of digital sampling of a physiologic signal. *Electroencephalography and Clinical Neurophysiology/Evoked Potentials* Section 89, 349-358.

MacCabee, P. J., & Hassan, N. F. (1992). AAEM minimonograph #39: Digital filtering: Basic concepts and application to evoked potentials. *Muscle & Nerve: Official Journal of the American Association of Electrodiagnostic Medicine*, *15*(8), 865-875.

Barry, D. T. (1991). AAEM minimonograph #36: basic concepts of electricity and electronics in clinical electromyography. *Muscle & Nerve: Official Journal of the American Association of Electrodiagnostic Medicine*, *14*(10), 937-946.

**Signal Processing (more applied, with coding examples)**

***Books***

Semmlow, J. (2011). *Signals and systems for bioengineers: a MATLAB-based introduction.* Academic Press.

Chaparro, L., & Akan, A. (2018). *Signals and Systems using MATLAB*. Academic Press.

**Mathematics (Advanced Topics)**

Papoulis, A., & Pillai, S. U. (2002). *Probability, random variables, and stochastic processes*. Tata McGraw-Hill Education.

Brillinger, D. R. (1981). *Time series: data analysis and theory* (Vol. 36). Siam.

Bendat, J. S., & Piersol, A. G. (2011). *Random data: analysis and measurement procedures* (Vol. 729). John Wiley & Sons.

**Instrumentation**

***Books***

Webster, J. G., & Eren, H. (Eds.). (2018). *Measurement, Instrumentation, and Sensors Handbook: Two-Volume Set*. CRC press.

***Instrumentation and Measurement: Guides for Clinicians***

Merletti, R., & Cerone, G. L. (2020). Tutorial. Surface EMG detection, conditioning and pre-processing: best practices. *Journal of Electromyography and Kinesiology*, 102440.

Tankisi, H., Burke, D., Cui, L., de Carvalho, M., Kuwabara, S., Nandedkar, S. D., ... & Fuglsang-Frederiksen, A. (2020). Standards of instrumentation of EMG. *Clinical neurophysiology*, *131*(1), 243-258.

Gitter, A. J., & Stolov, W. C. (1995). AAEM Minimonograph #16: Instrumentation and measurement in electrodiagnostic medicine–Part I. *Muscle & Nerve: Official Journal of the American Association of Electrodiagnostic Medicine*, *18*(8), 799-811.

Gitter, A. J., & Stolov, W. C. (1995). AAEM Minimonograph #16: Instrumentation and measurement in electrodiagnostic medicine–Part II. *Muscle & Nerve: Official Journal of the American Association of Electrodiagnostic Medicine*, *18*(8), 812-824.

Besomi, M., Hodges, P. W., van Dieën, J., Carson, R. G., Clancy, E. A., Disselhorst-Klug, C., ... & McGill, K. (2019). Consensus for experimental design in electromyography (CEDE) project: Electrode selection matrix. *Journal of Electromyography and Kinesiology*, *48*, 128-144.
