# Supplementary material for: Analysis and Biophysics of Surface EMG for Physiotherapists and Kinesiologists: Toward a Common Language With Rehabilitation Engineers
Source: Front Neurol. 2020 Oct 15;11:576729. doi: 10.3389/fneur.2020.576729 (PMC7594523; doi:10.3389/fneur.2020.576729)
Supplement: Supplementary Data Sheet 2 — Appendix: Advanced topics. [file Data_Sheet_2.DOCX]

# Appendix

This appendix covers more advanced signal processing concepts that are not essential material, but which may provide a more complete understanding of EMG signal analysis techniques.

## Power Spectral Density Estimation – Welch’s Method

To obtain a smoother power spectrum representation of the EMG signal, the total signal length can be divided into short segments or epochs containing a fixed number of samples, *L*. The number of samples in each EMG signal section or epoch (*L*) depends on the sampling frequency (*fs*, in samples/s or Hz) and the epoch length (*T_r_*, in seconds), Equation 1. Note that the length of the signal segment or epoch (*T_r_*) determines the “frequency resolution” of the Fourier-transformed signal and the lowest detectable frequency component (1/*T_r_*), Equation 2.

1. $T_{r} = \frac{L}{fs}$
2. $Frequency resolution (\Delta R) = \frac{1}{T_{r}}$

The power spectral density can then be estimated for each signal epoch, and these local estimates averaged to obtain the power spectral density of the entire signal length. One averaging approach for power spectral density estimation is Welch’s method (see **pwelch** function in MATLAB), which uses overlapping segments of the signal (which are multiplied by a window function) to obtain the power spectral density estimate, Figure 9. To obtain the average power spectral density, the total signal (containing *N* samples) is divided into a number of segments (*K*), where each successive segment starts *D* number of samples after the previous segment. When *K* is an integer value (whole number), the number of segments contained within the signal depends on *D* and the length of each segment in samples (*L*):

1. $K= N_{AVG} = \frac{N-L}{D}+1$

Larger values of *L* will give a better frequency resolution (*∆R* = *1*/*L*), see Section 3.2. However, larger values of *L* will also decrease the number of segments *K* and the number of averages performed (*N_AVG_*). Using Welch’s method thus requires a trade-off between frequency resolution and spectral stability (the amount of variance or noisy peaks in the spectrum), Figure 9 (e)-(f).

## Zero-padding

The discrete Fourier Transform (DFT) of a number of samples (*L*) taken from a continuous signal can be computed using fast Fourier transform (FFT) algorithms on several programming platforms (see function **fft** in MATLAB and Octave). The number of points used in the DFT (NFFT) should be greater than *L*, NFFT is commonly set to the next power of 2 greater than the original number of samples for computational speed (i.e. if *L* = 200, then NFFT is set to 256 or 2^8^). If NFFT is greater than the signal length, the original signal will be “zero-padded”, which simply means that zeros are added to the beginning or end of the time-domain signal to increase its length, Figure A1 (b). Zero-padding in the time-domain translates to interpolation in the frequency domain, i.e. increasing NFFT will increase the number of frequency bins (“frequency bins” can also be referred to as “spectral lines”) in the signal spectrum, Equation 4. Decreasing the spacing between frequency bins, ∆R_NFFT,_ in the signal spectrum can improve the accuracy of estimates of the spectral lines, Figure A1. It is important to note that although zero-padding can reveal important information, it does not create new information or change the frequency resolution of the underlying signal. For example, if both a 30 Hz and a 32 Hz sine wave were present in the signal in Figure A1, it would not be possible to distinguish (or resolve) distinct peaks for both signals in the frequency domain, as their difference in frequency is less than ∆R = 4 Hz. In order to resolve the 30 Hz and 32 Hz frequency components and correctly identify their amplitudes, the length of the signal would need to be increased to at least 0.5 s (∆R = 2 Hz, see Example (vi) in Tutorial Code). The spacing between consecutive frequency bins (∆R_NFFT_) in the power spectrum is determined by Equation 4, where *fs* is the sampling frequency and *NFFT* is the number of points in the DFT.

1. $\Delta R_{NFFT} = \frac{fs}{NFFT}$


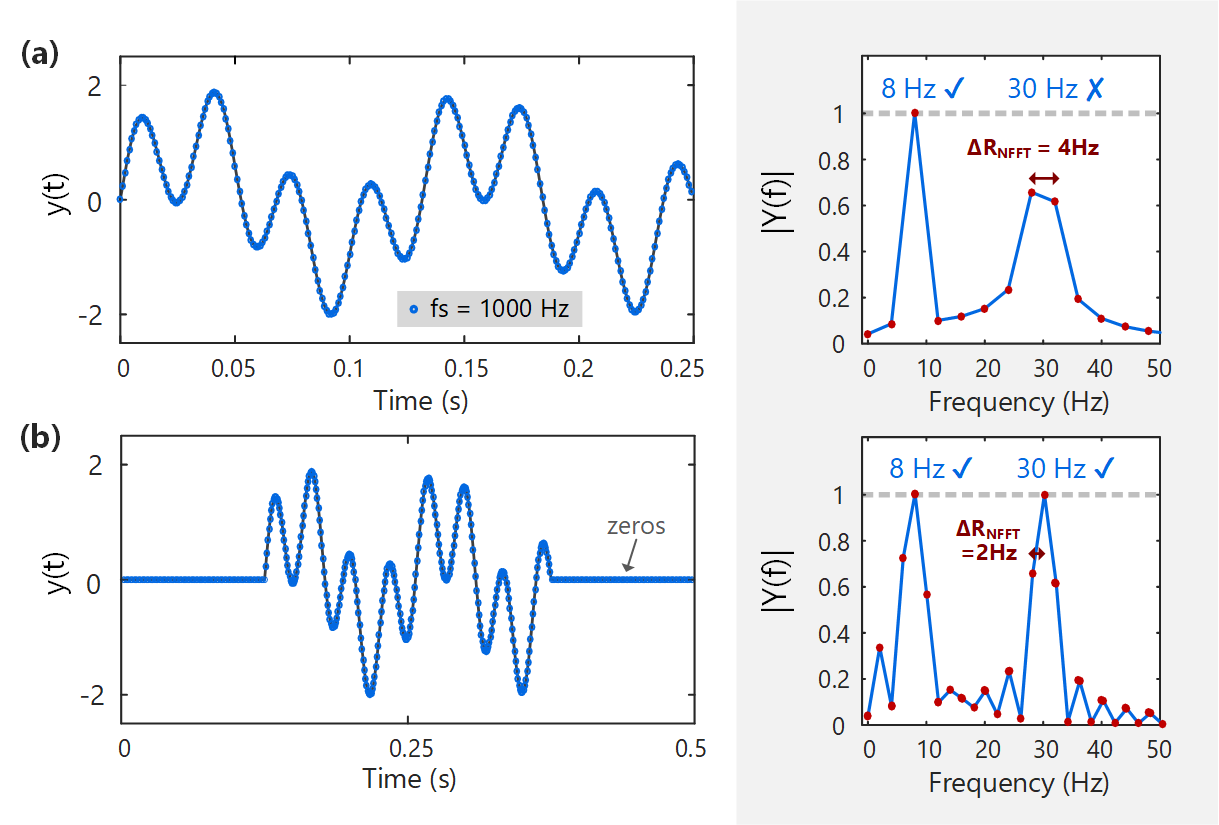


***Figure A1.*** *(a) A waveform that consists of an 8 Hz sine wave and a 30 Hz sine wave sampled at a rate (fs) of 1000 samples/s. The signal length is 0.25 s, i.e. 250 samples, resulting in a frequency resolution (∆R) of 4 Hz (note without zero-padding, ∆R = ∆R_NFFT_). The amplitude of the 8 Hz sine wave is correctly identified in the frequency domain as it is an integer multiple of 4 Hz (i.e. 8 is divisible by 4 with no remainder). However, the amplitude of the 30 Hz sine wave is not correctly estimated as it lies between the 28 Hz and 32 Hz bins. (b) By zero-padding the signal in the time-domain, the length of the NFFT is now 500 samples, ∆R is still 4 Hz but ∆R_NFFT_ =2 Hz (Equation 2 and Equation 4). As 30 Hz is an integer multiple of 2 Hz, its amplitude is now correctly estimated. See Examples (v) and (vi) in Tutorial Code.*

## Coherence

Surface EMG is often recorded from multiple muscles during single contraction, e.g. from multiple leg muscles active during gait. The linear correlation or dependency between two simultaneously recorded sEMG signals at each frequency can be estimated by calculating the coherence between the signals (see Equations in Terminology Matrix in the CEDE project ([Hodges, 2020](#_ENREF_5)), see function **mscohere** in MATLAB and Example (xviii) in Tutorial Code). Coherence can be used to reveal the coupling between EMG signals at specific frequencies and find the frequencies “common” to both signals. Corticomuscular coherence calculated between EEG, magnetoencephalographic (MEG)^[[1]](#footnote-1)^ and sEMG signals is similarly used to identify coupling between electrical signals generated by brain and muscle ([Conway et al., 1995](#_ENREF_3)). The magnitude of coherence ranges from 0 to 1 at each frequency, Figure A2 (c), with 0 indicating no correlation/relationship between the signals at a particular frequency and 1 indicating that the signals are completely “coherent” (i.e. have a perfect linear relationship). Coherence can also be estimated between individual motor unit firing times (decomposed from EMG signals recorded intramuscularly or from sEMG grids/arrays) to investigate the correlation between motor unit discharges at specific frequencies. Coherent motor unit firing in the beta frequency range (15 – 35 Hz) is directly correlated with short-term motor unit synchronization ([Lowery et al., 2007](#_ENREF_6); [McManus et al., 2015](#_ENREF_7)). However, it should be noted that two sEMG signals contaminated by noise or interference from the same source may also exhibit significant coherence (see also cross-talk). Further details on coherence can be found in [Carter (1993](#_ENREF_2)), [Halliday and Rosenberg (1999](#_ENREF_4)) and [Brillinger (1981](#_ENREF_1)).

*
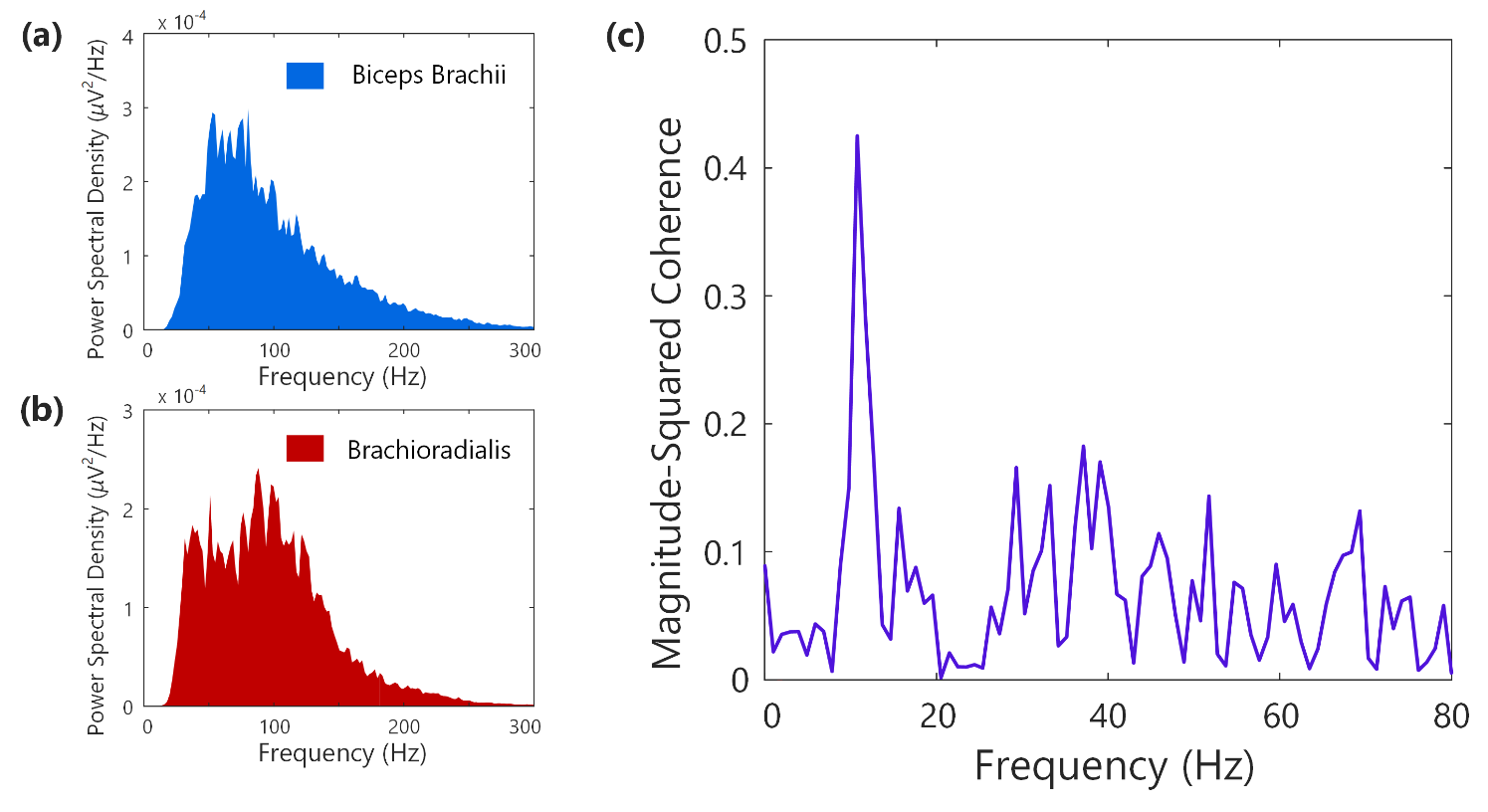
*

***Figure A2.*** *The power spectral densities of two sEMG signals simultaneously recorded from (a) the biceps brachii and (b) the brachioradialis during elbow flexion at 30%MVC. (c) The magnitude-squared coherence spectrum reveals the correlation between the two simultaneously recorded signals at each frequency. The EMG signals are correlated at ~11 Hz, represented by a peak in the coherence spectrum at this frequency. Note that an appropriate significance threshold should be chosen when determining whether detected coherence peaks are significant. See Example (xviii) in Tutorial Code.*

# References

Brillinger, D.R. (1981). *Time series: data analysis and theory.* Siam.

Carter, G.C. (1993). *Coherence and time delay estimation: an applied tutorial for research, development, test, and evaluation engineers.* IEEE.

Conway, B., Halliday, D., Farmer, S., Shahani, U., Maas, P., Weir, A., and Rosenberg, J. (1995). Synchronization between motor cortex and spinal motoneuronal pool during the performance of a maintained motor task in man. *The Journal of physiology* 489**,** 917-924.

Halliday, D.M., and Rosenberg, J.R. (1999). "Time and frequency domain analysis of spike train and time series data," in *Modern techniques in neuroscience research*. Springer), 503-543.

Hodges, P.W. (2020). Consensus for Experimental Design in Electromyography (CEDE) project. *Journal of electromyography and kinesiology: official journal of the International Society of Electrophysiological Kinesiology* 50**,** 102343.

Lowery, M.M., Myers, L.J., and Erim, Z. (2007). Coherence between motor unit discharges in response to shared neural inputs. *Journal of neuroscience methods* 163**,** 384-391.

McManus, L., Hu, X., Rymer, W.Z., Lowery, M.M., and Suresh, N.L. (2015). Changes in motor unit behavior following isometric fatigue of the first dorsal interosseous muscle. *Journal of neurophysiology* 113**,** 3186-3196.

1. EEG and MEG record the electrical and magnetic fields, respectively, generated by neuronal activity of the brain. [↑](#footnote-ref-1)
